# Toward Multiscale Models of Cyanobacterial Growth: A Modular Approach

**DOI:** 10.3389/fbioe.2016.00095

**Published:** 2016-12-26

**Authors:** Stefanie Westermark, Ralf Steuer

**Affiliations:** ^1^Fachinstitut für Theoretische Biologie (ITB), Institut für Biologie, Humboldt-Universität zu Berlin, Berlin, Germany

**Keywords:** photosynthesis, cyanobacteria, whole-cell models, flux balance analysis (FBA), circadian clock, CO_2_-concentrating mechanisms (CCMs), network reconstruction, metabolism

## Abstract

Oxygenic photosynthesis dominates global primary productivity ever since its evolution more than three billion years ago. While many aspects of phototrophic growth are well understood, it remains a considerable challenge to elucidate the manifold dependencies and interconnections between the diverse cellular processes that together facilitate the synthesis of new cells. Phototrophic growth involves the coordinated action of several layers of cellular functioning, ranging from the photosynthetic light reactions and the electron transport chain, to carbon-concentrating mechanisms and the assimilation of inorganic carbon. It requires the synthesis of new building blocks by cellular metabolism, protection against excessive light, as well as diurnal regulation by a circadian clock and the orchestration of gene expression and cell division. Computational modeling allows us to quantitatively describe these cellular functions and processes relevant for phototrophic growth. As yet, however, computational models are mostly confined to the inner workings of individual cellular processes, rather than describing the manifold interactions between them in the context of a living cell. Using cyanobacteria as model organisms, this contribution seeks to summarize existing computational models that are relevant to describe phototrophic growth and seeks to outline their interactions and dependencies. Our ultimate aim is to understand cellular functioning and growth as the outcome of a coordinated operation of diverse yet interconnected cellular processes.

## 1 Introduction

Almost all life on our planet ultimately depends on harvesting the light energy provided by the sun and the subsequent conversion of atmospheric CO_2_ and other inorganic nutrients into the building blocks of life. As one of the key inventions in evolution, oxygenic photosynthesis has transformed life on Earth and dominates the Earth’s primary productivity today (Lane, 2002; Morton, 2009). Beyond their evolutionary and ecological importance, phototrophic organisms are an essential resource for humankind and provide almost all food, feed, and fiber required to sustain human life on this planet with more than 7 billion inhabitants. Many of our strategies to master the challenges of the 21st century will inevitably rely on the growth of phototrophic organisms. Making better use of the sun’s light energy while avoiding past mistakes of industrial agriculture related to water usage, energy expenditure, eutrophication, and land use are necessary steps for a sustainable future.

Phototrophic microorganisms, in particular cyanobacteria, hold great promise as a renewable resource. Cyanobacteria are able to grow with high yield under adverse conditions and their cultivation does not rely on traditional farmland or fresh water. To make use of the biotechnological potential of cyanobacteria, however, requires further understanding of the organization of phototophic growth. While many aspects of phototrophic growth are well known and many details of photosynthetic functioning have been unraveled by decades of active research, it still remains a considerable challenge to understand the individual cellular processes in the context of a living cell.

To this end, the construction of computational models of cellular processes offers the possibility to investigate the emergent properties that arise from interacting processes. Corresponding to the path of experimental research, however, to date almost all computational models involving cyanobacterial functioning and growth focus on the inner workings of individual processes, such as the path of electrons in photosystem II or the functioning of the circadian clock. But phototrophic growth is an organismic property. It is not so much an individual process that gives rise to cellular growth, rather it is the interplay of individual processes that bears reproduction and growth of living cells.

In this contribution, we seek to provide an overview on processes relevant to cyanobacterial growth and summarize the available computational models thereof. Our aim is not encyclopedic, that is, we do not aim for a comprehensive account of all available models. Rather, we seek to focus on representative models that may contribute to our understanding of the functioning of a cell as a whole. Our focus are also not the, albeit important, minutiae of individual processes and models, but rather how they can be collated into a coherent whole. Our starting point is a set of existing computational descriptions of cellular processes and their possible interactions. Our ultimate goal is to describe cellular adaptation, cellular resource allocation, and phototrophic growth in complex environments. Or, as more eloquently put by Neidhardt (1999) already more than 15 years ago: “We must solve the cell. That is, we must do our best to design a computer-based model that can predict overall cell behavior for steady states of growth and for transitions between steady states. The model will at first be crude, inaccurate, and a complete failure at some tasks. With increasing refinement based on additional experimental data, the model should gradually improve. Importantly, the model will guide experimental inquiry by indicating areas of inadequate, insufficient, or incorrect information. Vitally, it is only through such modeling of whole-system behavior—that is, of growth—that one will learn how near and how far our knowledge takes us toward understanding the living cell.”

Our premise is that sophisticated computational models are already available for many of the processes that underlie phototrophic growth. Modeling their interactions, however, is still no trivial task. First, most models focus on the inner workings of the processes they describe—and therefore often do not describe key variables that govern the interaction with other processes. Second, the various subprocesses and time scales involved in a computational description of phototrophic growth typically require the use of different mathematical and computational concepts, which cannot always be easily reconciled within a single computational description. We seek to summarize these different computational descriptions and aim to highlight common variables and interactions. Importantly, we do not necessarily aim at a single unified model that encompassed all aspects of a growing cell. Rather, we argue for a modular approach—a growing set of models that describe aspects of cyanobacterial growth on different temporal and spatial scales. Depending on the research question, and the temporal and spatial scales involved in this particular research question, different descriptions of cyanobacterial functioning may be chosen—and utilized to derive the emergent properties of cellular growth by putting the parts together.

## 2 Modeling Phototrophic Growth: An Overview

**Figure 1:**
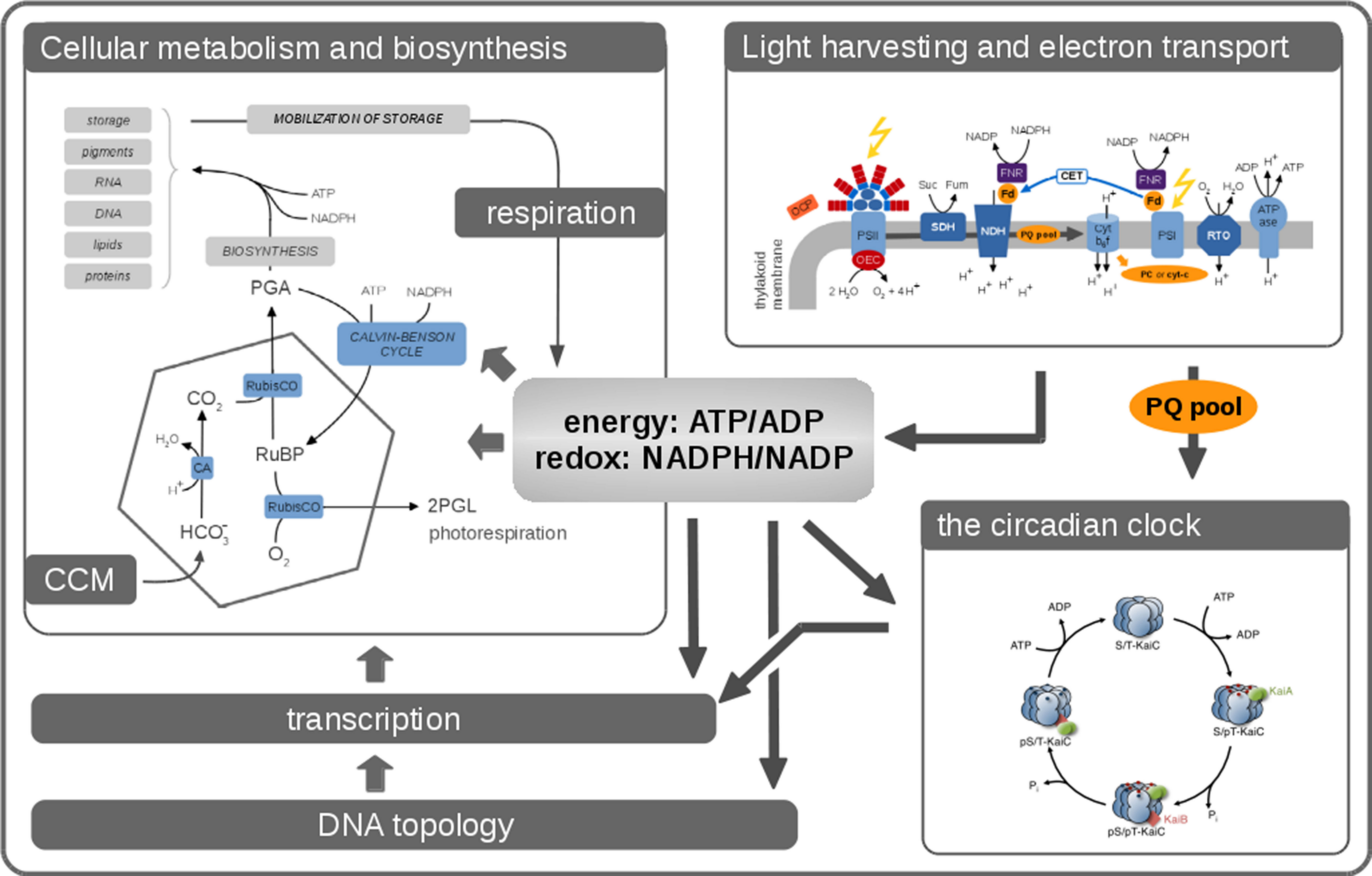
**Phototrophic growth is an emergent property that arises from interacting cellular processes**. Cellular growth can be described by considering these processes as interacting modules. Many aspects related to cellular growth are reasonably well understood, and detailed computational models already exist. Nonetheless, it remains a considerable challenge to integrate these diverse models into a coherent whole. In this contribution, we seek to describe the processes that are relevant for cyanobacterial functioning and growth. Key aspects are the photosynthetic light reactions, providing energy and reductants for cellular metabolism, cellular respiration in the absence of light, CO_2_-concentrating mechanisms (CCMs), transcription, and global energy-induced changes in DNA topology, as well as the circadian clock and its integration into diverse layers of cellular regulation.

Cellular growth is an organismic process that arises from a coordinated interplay of cellular functions. In the following, we briefly describe the key processes relevant to cyanobacterial functioning and growth in complex environments. An overview is provided in Figure 1.

Survival and growth of (most) cyanobacteria begins with the absorption of photons facilitated by large light-harvesting antennae, the phycobilisomes, and chlorophyll *a*. The energy harvested from sunlight drives water splitting at photosystem II (PSII). Electrons, derived from water, are provided to the electron transport chain (ETC) and molecular oxygen is released as the byproduct of photosynthesis.

The ETC consists of a number of large protein complexes, mostly located in the thylakoid membrane. Electrons are transferred along the ETC, ultimately resulting in the regeneration of adenosine triphosphate (ATP) and reduced nicotinamide adenine dinucleotide phosphate (NADPH) as energy carrier and reductant, respectively. The functioning of the photosystems and the ETC are complex biophysical processes and objects of intense research. The respective processes are characterized by fast time scales and transitions between a large number of possible states. While a number of detailed computational models of these processes are available, often with a focus on photosystem II, the respective models typically do not describe regeneration of ATP and NADPH, and hence are not straightforwardly connected to other cellular functions.

The ATP and NADPH regenerated by the photosynthetic light reactions play a crucial role for almost all other cellular processes. Beyond their role as energy donor and reductant, they also serve as important signaling compounds to convey information about the intracellular state. Regenerated ATP and NADPH are utilized to assimilate atmospheric carbon dioxide (CO_2_). Cyanobacteria possess mechanisms to concentrate inorganic carbon in the vicinity of the CO_2_-fixing enzyme, the ribulose-1,5-bisphosphate carboxylase/oxygenase (RuBisCO), making use of bacterial microcompartments known as carboxysomes. Compared to the light reactions, the relevant time scales of the so-called dark reactions are significantly slower. Modeling of CO_2_-concentrating mechanisms (CCMs) typically involves consideration of diffusion and spatial structure.

The carbon assimilated by the enzyme RuBisCO serves as a substrate to synthesize new cell components, including storage compounds and substrates for cellular respiration. Cellular metabolism involves several hundreds of biochemical reactions, catalyzed by enzymes, as well as spontaneous interconversions, transport, and diffusion processes. From a computational perspective, a description of cellular metabolism must involve several spatial and temporal scales. Detailed models for the action of individual enzymes, such as RuBisCO, exist, based on detailed enzyme mechanisms and elementary reaction steps. Pathways are typically described by combining, often approximative, kinetics of the involved enzymes into larger kinetic models, described by ordinary differential equations (ODEs).

While current kinetic models of metabolism rarely involve more than a few dozen compounds, cellular metabolism is increasingly analyzed using large-scale metabolic reconstructions and constraint-based computational methods. Metabolic network reconstructions are based upon the predicted gene content deduced from genomic DNA and aim to provide an unbiased and comprehensive account of all interconversions of small molecules inside a single cell or a compartment. Unlike kinetic models, metabolic reconstructions only make use of the stoichiometric properties of the respective interconversions, and manually curated reconstructions have been reported for a number of cyanobacteria (Knoop et al., 2010, 2013; Montagud et al., 2010; Nogales et al., 2012; Saha et al., 2012; Vu et al., 2012; Yoshikawa et al., 2015). Highly efficient computational methods exist that allow for the analysis of networks that consist of several hundreds of biochemical reactions and other molecular interconversions. These computational methods, however, are challenging to reconcile and integrate into more traditional enzyme kinetic models of metabolism.

In addition to the photosynthetic light reactions and cellular metabolism, cyanobacterial functioning also involves a large number of regulatory processes. Most prominent is the cyanobacterial circadian clock. Unique among all known prokaryotes, cyanobacteria possess a true circadian clock, a self-sustained oscillator that is entrained to an external *zeitgeber*. Since its discovery in the late 1980s, the cyanobacterial circadian clock has been an object of intense research (Pattanayak and Rust, 2014). Early research, however, was mostly focused on the inner workings of the clock, the molecular details of the core clock, and its input pathways. Only recently, interactions between the clock and metabolism and the question how the clock functions within a broader cellular context have been addressed in more detail (Pattanayak and Rust, 2014; Diamond et al., 2015; Shultzaberger et al., 2015).

Correspondingly, a number of quantitative computational models exist that describe the mechanistic details of the cyanobacterial clock, as well as its entrainment to environmental cues—but as yet only few of these models allow for a straightforward integration into a broader cellular context. While there is increasing evidence how the clock is influenced by, and itself influences, photosynthetic light reactions and metabolism, via sensing metabolic activity (Pattanayak et al., 2015) and redox state (Kim et al., 2012) and controlling transcription regulation, the precise evolutionary role of the clock remains insufficiently understood. Elucidating how the circadian clock interacts with other cellular processes and to integrate models of the cyanobacterial circadian clock into a broader cellular context, with the aim to understand how timing mechanisms affect cellular fitness, is a timely question for further computational research.

Energy metabolism and growth, like all cellular processes, are also dependent on the transcriptional and translational machinery. The transcriptional landscape of commonly cultivated cyanobacteria, such as *Synechocystis* sp. PCC 6803, is increasingly known (Kopf et al., 2014) and a number of studies investigated transcriptional rhythms in the presence of light–dark phases (Lehmann et al., 2013; Beck et al., 2014). Of particular interest is also the role of small regulatory RNA (sRNA) to coordinate cellular processes. For several cyanobacterial strains, most notably *Synechocystis* sp. PCC 6803, substantial sRNA transcription, intragenic transcripts, and antisense transcripts have been reported (Kopf and Hess, 2015). While generic computational models for various possible role of regulatory RNA exist (Legewie et al., 2008), these are currently not integrated within larger computational efforts to understand growth properties and adaptation of cyanobacteria.

As a key driver of cellular functioning and growth, global gene expression is believed to be under direct control of the circadian oscillator, mediated by the topological properties of the cyanobacterial chromosomes. It was shown that the superhelicity of the DNA undergoes rhythmic changes that drive global changes in gene expression (Woelfle et al., 2007; Vijayan et al., 2009). It is further known that the rate of transcription also depends on the local supercoiling status of DNA. Vice versa, supercoiling depends on the cellular energy status, since the extent of supercoiling achieved by the DNA gyrase is strongly dependent on ATP hydrolysis. For heterotrophic organisms, specifically *Escherichia coli*, these observations have led to the proposal of homeostatic control and a feedback loop between the intracellular ATP/ADP ratio, DNA supercoiling, transcription, and again changes in the ATP/ADP ratio (Wijker et al., 1995). Closely related ideas have been put forward in the context of ultradian rhythms in yeast where global partitioning of anabolism and catabolism might be mediated by ATP feedback loop on chromatin architecture (Amariei et al., 2013). In the case of cyanobacteria, a global feedback between cellular energy state, DNA supercoiling, and transcription might mediate between global transcription rhythms, the light reactions as the source of cellular energy, and the circadian clock. Such global feedbacks are currently not explicitly considered in models of cyanobacterial growth and are challenging to implement because of the diverse layers of cellular regulation involved.

Parallel to the efforts of molecular biology to understand the mechanistic and biophysical basis of the processes involved in phototrophic growth, there is a rich history of phenomenological phytoplanktonic growth models. Phenomenological growth formulations are typically employed in models of marine ecosystems and food webs, as well as biogeochemical models to understand the global response of ecosystems to environmental changes. Phenomenological growth models often employ Monod-type equations to describe uptake of a limiting nutrient. Following the early work of Droop (1968), also more sophisticated approaches exist to describe variable internal quotas, see Droop (1983) for an overview. The dynamics of simple phytoplankton growth models are typically based on empirical parameter fitting, rather than an outcome of the underlying cell physiology, and involve strong simplifications, such as using a constant carbon-to-nitrogen (C:N) stoichiometry and absence of photoacclimation (Ayata et al., 2013). It has been pointed out that a major shortcoming of such models is their limited ability to produce true emergence in marine ecosystem models (Allen and Polimene, 2011). Specifically, these models do not evolve to new states not already incorporated in their formulation that makes them unsuitable to properly predict ecosystem changes under changing environmental conditions. As argued by Allen and Polimene (2011), the path forward is to place more emphasis on the underlying intracellular processes—resulting in physiological growth formulations that allow for trade-offs between resource allocations of physiological activities, and hence the possibility to produce biogeochemical and ecological dynamics as emergent properties. Preliminary models, albeit still limited, that combine a detailed description of photosynthesis and phytoplankton growth are already available (Kroon and Thoms, 2006).

In the following, we seek to discuss selected computational models related to cyanobacterial functioning and growth in more detail. Our view is that cyanobacterial physiology depends on interacting cellular processes that can be interpreted as functional “modules”, such as the photosynthetic light reactions and the ETC, carbon uptake mechanisms, cellular metabolism, the circadian clock, as well as the transcriptional and translational machinery and its regulation. For many of these modules, reasonable computational descriptions already exist, whereas other processes, for example, the coordination of cell cycle events in relation to metabolism (Asato, 2005, 2006), have not yet been subject to computational studies.

**Figure 2:**
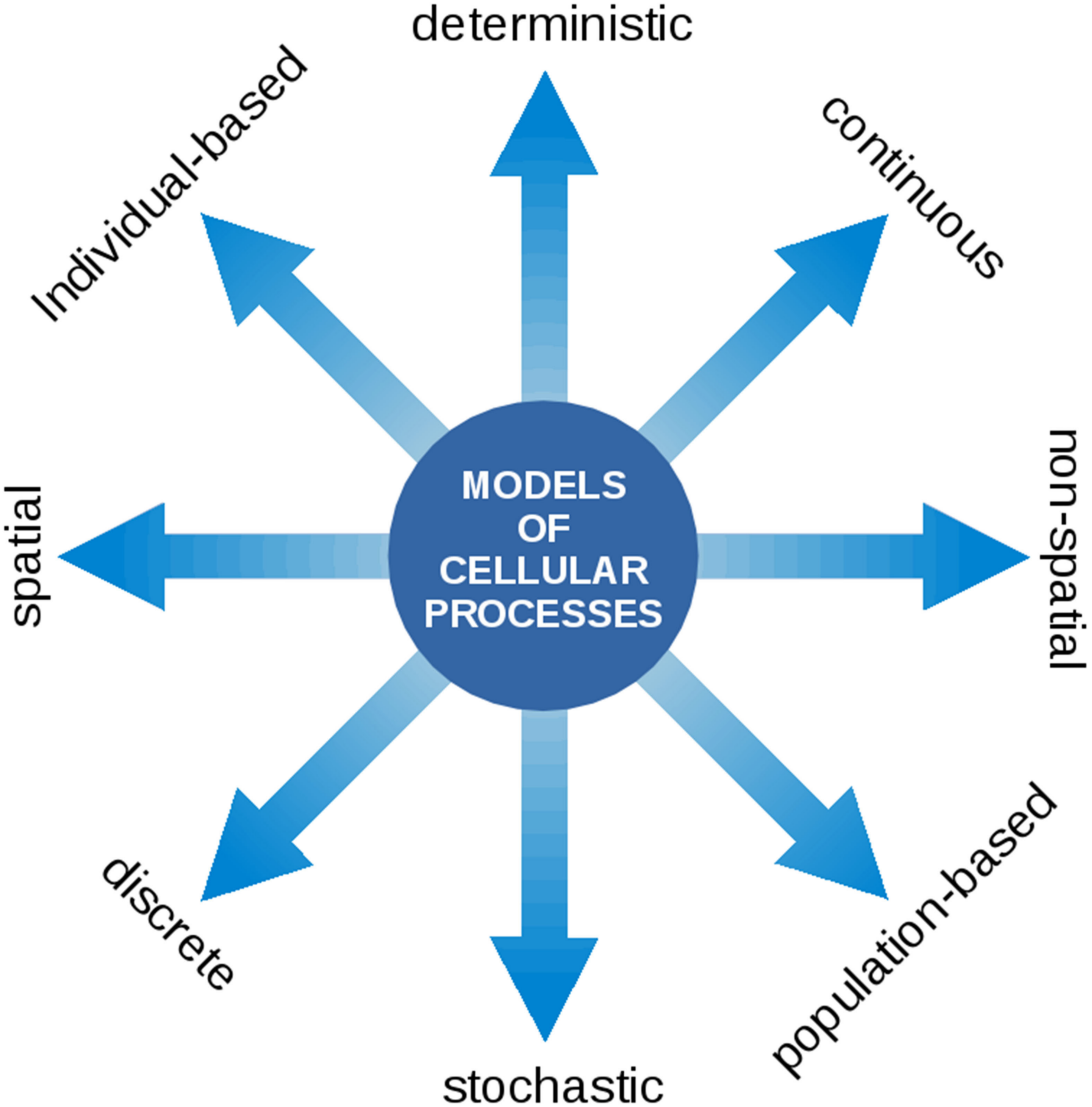
**Models of cellular processes are highly diverse and may involve a wide range of computational concepts and methodologies**. At the core of the modeling process is a translation of a biological processes into a formal (mathematical) language. Once this translation is established, the model can be interrogated using the tools of mathematical and computational analysis. The most prevalent representations of cellular processes described in this contribution make use of deterministic ordinary differential equations (ODEs) to describe the time-dependent dynamics of continuous intracellular concentrations, typically on the population level. In the following, such models are denoted as kinetic models and may either make use of heuristic approximate rate equations or rate equations derived from explicit biochemical mechanisms. Models of CCMs typically involve a spatial component and originate from a description based on partial differential equations (PDEs). Models of the light reactions frequently describe transitions between discrete states that occur with a certain state-dependent probability. Flux balance models consists of a set of linear relationships (linear inequality constraints) between variables and make use of linear programing (LP), a method to identify the optimum of a linear objective function. For a more detailed overview on model types, see also Steuer and Junker (2009).

Our aim is to highlight the common variables and known interactions between the processes relevant to cyanobacterial functioning and growth. In this respect, a particular challenge is the wide range of computational approaches and methods used. Models of cellular processes may take many forms, ranging from spatial versus non-spatial, stochastic versus deterministic, population level versus single cell level, and continuous versus discrete descriptions. See Figure 2 for an overview. Notwithstanding the technical challenges, we believe that the integration of different aspects of cellular growth, and their respective computational representation, is a prerequisite toward understanding the living cell. We seek to understand how phototrophic growth functions and how it is regulated. How does the coordination of physiological functions work in order to synthesize the right macromolecules at the right time? Which level of detail is required to describe cellular growth? What are the variables and time scales involved?

## 3 Models of the Photosynthetic Light Reactions

Phototrophic growth begins with the absorption of light and its conversion into chemical energy. Despite a number of open questions and the need for further research, many of the fundamental properties of oxygenic photosynthesis have been elucidated in the past century. Owing to the fact that cyanobacteria are the evolutionary ancestors of modern-day chloroplasts, the organization of their photosynthetic ETC is essentially identical to that in algae and green plants (Vermaas, 2001).

**Figure 3:**
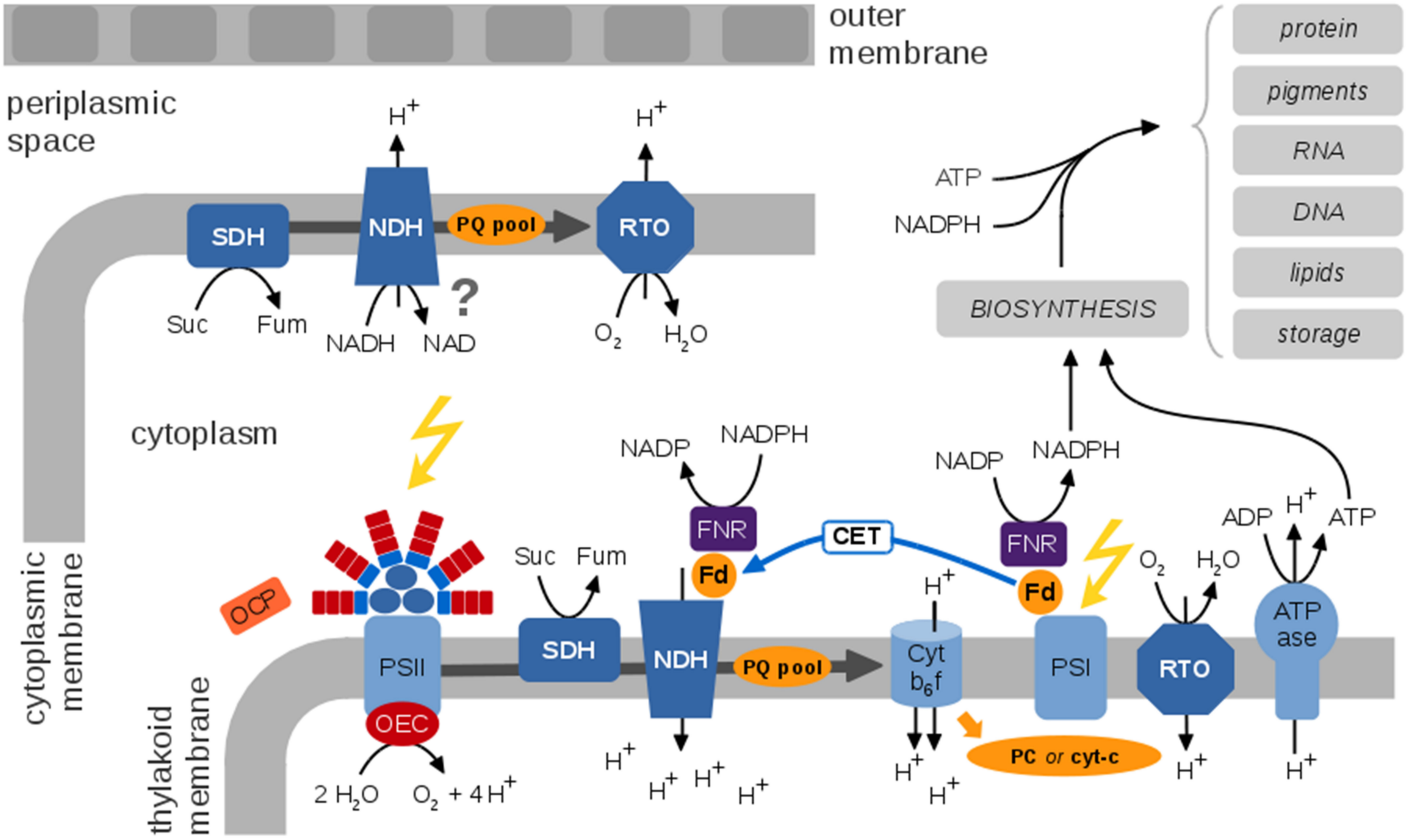
**A generic view of the cyanobacterial electron transport chains (ETCs), species-specific differences are neglected**. Cyanobacterial photosynthetic and respiratory ETCs share common components. The protein complexes of the photosynthetic ETC are embedded within the thylakoid membrane enclosing the thylakoid lumen. Key protein complexes are photosystem II (PSII), with the oxygen-evolving complex (OEC), the cytochrome b_6_f complex (Cytb_6_f), photosystem I (PSI), and the ATP synthase (ATPase). Cyclic electron transport involves the NDH complex and is still insufficiently understood. The Orange Carotenoid Protein (OCP) is a light sensor and energy quencher that interacts with the phycobilisomes to decrease energy arriving to the photosynthetic reaction centers (Kirilovsky and Kerfeld, 2013). The thylakoid membrane also contains respiratory components, in particular the succinate dehydrogenase (SDH), NADPH dehydrogenase (NDH), and a respiratory terminal oxidase (RTO). It has been suggested that ferredoxin (Fd) transfers electrons from NADPH to NDH-1 via the Fd-NADP^+^-reductase (FNR) (Ma and Ogawa, 2015). The cytoplasmic membrane contains a rudimentary respiratory electron transport chain; functional details and localization of putative components are largely unknown. Photosynthetic ETCs result in regeneration of ATP and NADPH for cellular synthesis and growth. A residual respiratory activity persists also in the presence of light. The photosynthetic and respiratory ETCs are subject to multiple alternative electron pathways (not shown) that act as “electron valves” to prevent overreduction of the ETC (Mullineaux, 2014).

In most cyanobacteria, light harvesting is facilitated by large antenna complexes, the phycobilisomes. Phycobilisomes are attached to the cytoplasmic surface of the thylakoid membrane (Mullineaux, 2014). The detailed composition of phycobilisomes is strain specific and depends on light quality, denoted as complementary chromatic adaptation. The energy absorbed by the phycobilisomes is transferred to either photosystem II or photosystem I, or dissipated as heat or fluorescence. The protein complexes of the photosynthetic ETC are embedded within the thylakoid membrane. The key proteins complexes responsible for photosynthetic electron transport are Photosystem II (PSII), the Cytochrome b_6_f complex (Cytb_6_f), Photosystem I (PSI), and ATP synthase (ATPase). See Figure 3 for an overview.

PSII splits water and reduces the plastoquinone (PQ) pool. The latter mediates the transport of electrons from PSII to Cytb_6_f. At Cytb_6_f, electrons are transferred to a soluble electron carrier on the luminal side of the thylakoid membrane, either plastocyanine (PC) or cytochrome-*c* (cyt-*c*). At PSI, electrons are transferred to ferredoxin and eventually to NAPDH using light-induced excitation of the PSI reaction center (linear electron transport, LET). Alternatively, electrons from the excited PSI state can be transferred back to PQ and Cytb_6_f (cyclic electron transport, CET), details of CET are still under debate and insufficiently understood. Photosynthetic electron flow results in a protein gradient across the thylakoid membrane that drives regeneration of ATP by the ATPase.

Unique to cyanobacteria, as opposed to plants and microalgae, is the combination of oxygenic photosynthesis and respiration in the same membrane system using intersecting ETCs and common components (Vermaas, 2001). The respiratory ETC involves the succinate dehydrogenase (SDH), the NADPH dehydrogenase (NDH-1), and terminal oxidases. The PQ pool, the Cytb_6_f complex, and PC (or cyt-*c*) as soluble electron carrier are involved in respiratory as well as photosynthetic electron transport. While photosynthesis exclusively takes place in the thylakoid membrane, a rudimentary respiratory chain is also present in the plasma membrane (Schultze et al., 2009).

**Figure 4:**
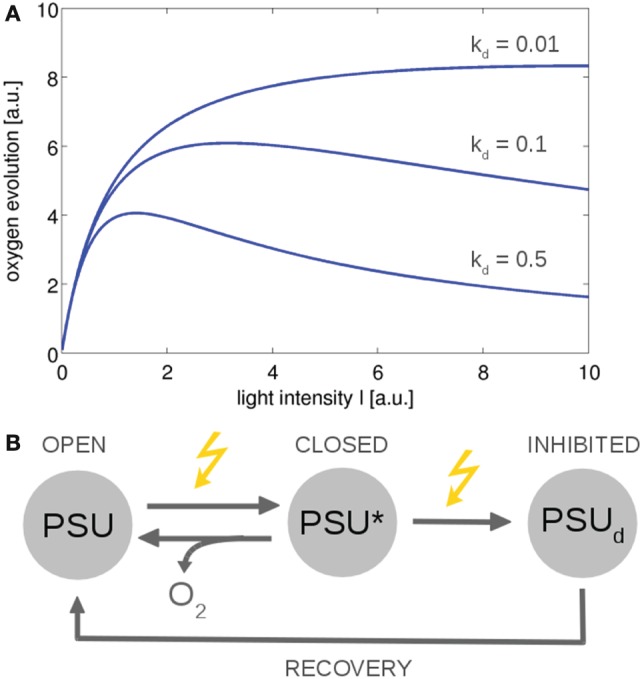
**The photosynthesis–irradiance (PI) curve for a minimal model of photosynthesis (Han, 2002)**. The ETC is described by a photosynthetic unit (PSU) that exists in an open or reactive state. After being subjected to light, the PSU transits to a closed or activated state (PSU*). Excessive absorption results in photodamage and an inhibited state PSU*_d_* with rate constant *k_d_*. **(A)** Using simple ODEs based on mass action kinetics results in typical PI curves. The overall functional form is similar to the Haldane equation, an equation derived for enzymes whose substrates have an inhibitory effect at higher concentrations. **(B)** The reaction scheme. Similar models can describe non-photochemical quenching in which the damaged state corresponds to a quenching state (indirectly) activated by light (Gorbunov et al., 2011). All values are reported in arbitrary units (a.u.). The ODEs used to generate the figure are provided in the Appendix.

From a computational perspective, photosynthesis in cyanobacteria and microalgae can be described on different levels of complexity. Basic models are closely related to overall growth models in ecology—and typically to reproduce the production of oxygen and the photosynthesis–irradiance (PI) curve of cyanobacteria and microalgae. Early models were derived by Crill (1977), Megard et al. (1984), Eilers and Peeters (1988), and Zonneveld (1998) among others. These models make use of a highly simplified photosynthetic factory or photosynthetic unit (PSU) that encompasses PSII, PSI, and the ETCs. See Figure 4 for an example. The resulting differential equations for the dependency of photosynthesis on light intensity can often be solved analytically, with a solution analogous to the Haldane equation—an enzyme kinetic equation that was derived for substrates with inhibitory effects at high concentrations. Simple three-state models are suitable to describe basic features of photoinhibition and the PSII repair cycle (Tyystjärvi et al., 1994). In later iterations, the parameters of the basic three-state model were augmented with a more mechanistic interpretation (Han, 2001, 2002), and the models were extended to describe the effects of intermittent light (Rubio et al., 2003). Recently, a basic three-state model was also applied to describe the kinetics of non-photochemical quenching (NPQ), induced by an orange cartenoid protein (OCP), in cyanobacteria (Gorbunov et al., 2011). To this day, simple three-state models remain relevant to describe overall photosynthetic activity, in particular in bulk models to assess productivity in photobioreactors and related industrial application (Nedbal et al., 2010; Bernard, 2011). With respect to understanding interactions between cellular processes, a drawback of highly simplified growth models is their insufficient representation of intracellular parameters, such as no explicit PQ pool, no explicit regeneration of ATP and NADPH, and lack of alternative electron transport.

Beyond overall bulk models of photosynthesis, there is a significant history of biophysical models to understand oxygen evolution and chlorophyll fluorescence transients, often with a focus on PSII, as well as to understand specific properties, such as energy distribution in the photosynthetic apparatus (Butler and Strasser, 1977; Butler, 1978) or, more recently, excitation transfer in the PSII membrane (Amarnath et al., 2016). Early kinetic models were described by Mar and Govindjee (1972), a more elaborate model was put forward by Holzwarth et al. (2006) and later analyzed by Nedbal et al. (2007). Further elaborate models of this kind were developed by Lazár (2003) and Zhu et al. (2005). The former was refined and extended by Jablonský and Lazár (2008); different approaches were later compared by the same authors (Lazár and Jablonský, 2009). Common to these models is a focus on chlorophyll fluorescence emission, and to a lesser extent oxygen evolution, as the main output variables. While relevant for biophysical research, the respective models cannot be straightforwardly integrated into more comprehensive models of phototrophic growth, due to the focus on fast time scales and specific output variables. We note that the interpretation of results obtained from pulse-amplitude modulated (PAM) fluorimetry significantly differs between cyanobacteria and plants (Schuurmans et al., 2015; Acuña et al., 2016), with modeling approaches focusing almost exclusively on the latter.

Models that explicitly describe the photosynthetic electron transport chain and subsequent reactions, in addition to PSII, are more suitable to integrate into the context of a living cell. To this end, a small number of models exist (Berry and Rumberg, 2000; Vershubskii et al., 2014), typically based on ODEs. An elaborate model of this type was proposed by Laisk et al. (2006), developed to understand the photosynthetic process from light absorption to sucrose synthesis. The model neglects many of the detailed biophysical properties of earlier models (Zhu et al., 2005), such as an explicit representation of the s-states that describe the cyclic reactions of the oxygen-evolving complex (Kok et al., 1970). The model instead provides a combination of whole chain electron transport and carbon assimilation processes, including non-photochemical quenching, chlorophyll fluorescence, and (albeit simplified) photorespiration. Using a similar approach, Zhu et al. (2013) described a detailed dynamic model of leaf photosynthesis, based on ODEs, from light capture to carbon assimilation, and incorporates the previous partial model of the same authors (Zhu et al., 2007) augmented by additional reactions of the ETC. Both models focus on C3 plant metabolism, but similar approaches are feasible for cyanobacteria.

Importantly, both models provide a sufficient level of detail to interface with other cellular processes and include ATP regeneration and reduction of NADPH, photorespiration, alternative electron transport, as well as an explicit representation of the PQ pool and lumenal pH. Owing to the focus on plant C3 metabolism, neither of the models describe peculiarities of cyanobacteria, such as shared components between the photosynthetic and respiratory ETC and the resulting differences in regulation.

Selected models of the ETC and the photosynthetic light reactions are summarized in Table 1. Main challenges for the development of corresponding models for cyanobacteria are to incorporate the respiratory ETC, as well as to incorporate the specific alternative electron sinks of cyanobacteria. As an interface to other cellular processes, discussed below, a model of the cyanobacterial ETC should include regulatory switches in cyanobacterial photosynthesis, regulation of light harvesting including regulation of the orange carotenoid protein, state transitions that control the relative energy transfer from phycobilisomes to PSII versus PSI, and alternative electron sinks that serve as “electron valves” and prevent overreduction of the ETC, among other features that are relevant for cyanobacterial functioning and growth (Mullineaux, 2014). Relevant exchange variables are ATP and NADPH regeneration, the state of the PQ pool, leakage of reactive oxygen species (ROS), and oxidation of metabolites for cellular respiration.

**Table 1:** **Selected models of the photosynthetic light reactions and the electron transport chain (ETC) in plants and cyanobacteria**.

Han (2002)	**A mechanistic model of algal photoinhibition induced by photodamage to photosystem II** A minimal three-state model describing the photosynthetic response to irradiance (PI) curve based on ordinary differential equations (ODEs). The model is representative of a family of similar models, including applications on non-photochemical quenching in cyanobacteria (Gorbunov et al., 2011).
Zhu et al. (2005)	**Chlorophyll a fluorescence induction kinetics in leaves predicted from a model describing each discrete step of excitation energy and electron transfer associated with Photosystem II** A detailed model of fluorescence induction in PSII in plant leaves based on ODEs. Table 3 in Zhu et al. (2005) provides a comparison of major assumptions and results of current models of fluorescence induction.
Laisk et al. (2006)	**C3 photosynthesis *in silico*** An integrated model of photosynthetic electron transport in C3 plants, including carbon reduction via the Calvin–Benson cycle based on ODEs. One of the first integrated models of the entire photosynthetic machinery.
Jablonský and Lazár (2008)	**Evidence for intermediate S-states as initial phase in the process of oxygen-evolving complex oxidation** A detailed model of PSII to describe oxygen evolution and chlorophyll fluorescence based on ODEs.
Jablonský et al. (2008)	**Impact of dimeric organization of enzyme on its function: the case of photosynthetic water splitting** An improved model of dimeric PSII describing the role of PSII dimerization on oxygen evolution. The model is based on ODEs and is an example of a very large number of variables (>10^3^) due to the combinatorial explosion of possible states and transitions between them.
Ebenhöh et al. (2011)	**A minimal mathematical model of non-photochemical quenching of chlorophyll fluorescence** A highly simplified model to describe light harvesting and short-term adaptive quenching dynamics in the plant photosynthetic ETC based on ODEs. Variables are the three states of the reaction centers, the oxidized and reduced PQ pool, lumenal proton concentration, active and inactive quencher, as well as stromal ATP and ADP concentrations.
Zhu et al. (2013)	**e-Photosynthesis: a comprehensive dynamic mechanistic model of C3 photosynthesis: from light capture to sucrose synthesis** A detailed model of leaf photosynthesis from light harvesting to carbohydrate synthesis based on ODEs. The model is a synthesis of the earlier model of Zhu et al. (2005) and a model of the core carbon metabolism (Zhu et al., 2007).

*This table and all following tables are not exhaustive but highlight selected models that are of particular relevance to describe aspects of cyanobacterial growth. Model details, such as the exact number of variables, are listed only if they can be unambiguously sourced from the original publication*.

## 4 Kinetic Models of Cellular Metabolism

The energy harvested by the photosynthetic light reactions drives the assimilation of inorganic carbon and the synthesis of storage compounds and building blocks for cellular growth. Photoautotrophic metabolism involves the uptake of inorganic carbon facilitated by CO_2_-concentrating mechanisms (CCMs), assimilation of CO_2_ by RuBisCO, and the subsequent synthesis of cellular building blocks mediated by a network of metabolic reactions. Computational concepts used to describe cyanobacterial metabolism have been discussed previously (Steuer et al., 2012), here we focus on the integration of such descriptions into integrative models of phototrophic growth. In particular, models of metabolism are highly diverse and span multiple orders of magnitude with respect to the time scales and number of variables involved. As highlighted previously (Steuer and Junker, 2009), no single universal computational methodology exists that is suitable to describe all relevant aspects of metabolic functioning. Rather, a hierarchy of computational approaches exists, ranging from detailed kinetic models of individual enzymatic reactions, based on ODEs, to large-scale stoichiometric reconstructions that are evaluated using constraint-based analysis.

The basic building blocks of metabolism are the actions of individual enzymes and their respective reaction mechanisms. Computational modeling of enzyme kinetics is well understood (Steuer and Junker, 2009; Sauro, 2014), even though specific reaction mechanisms and atom transition maps are not yet comprehensively available and must be confirmed on an individual per-reaction basis. Detailed kinetic models of key reactions have been proposed in the literature, most notably for RuBisCO, the key enzyme of the Calvin–Benson cycle (Witzel et al., 2010). Following the rules for overall rate equations of enzyme kinetic mechanisms, multiple reaction steps can be integrated into larger models of cellular pathways. Corresponding detailed kinetic models of metabolic pathways exist since the late 1950s, see Steuer and Junker (2009) for a review, and several kinetic pathway models have since been proposed for phototrophic plant metabolism. Of particular interest is the Calvin–Benson cycle and the adjacent carbon metabolism. Among the first computational descriptions of phototrophic C3 carbon metabolism were the models proposed by Milstein and Bremermann (1979) and Hahn (1984). The former involves 17 first-order ODEs and 22 parameters. The latter involves 19 state variables and describes the dynamics of Calvin–Benson cycle intermediates, as well as parts of sucrose and starch metabolism. The model was later extended to include photorespiration (Hahn, 1987), and simplified representations were considered (Hahn, 1991). The latter analysis demonstrated that also smaller models are able to reproduce the observed dynamics. Both models, as well as the model of Laisk and Walker (1986), are largely based on mass action kinetics, rather than derived enzyme kinetic equations for kinetic mechanisms. Parallel to the development of detailed kinetic models of core carbon metabolism, a number of biochemical models of photosynthetic CO_2_ assimilation have been developed that focus on plant-specific properties, such as gas exchange and stomatal conductance, often also incorporating aspects of carbon reduction, see Farquhar et al. (1980) for an influential early example. Likewise, a significant number of models were developed to investigate the origin of photosynthetic oscillations (Giersch, 1986; Laisk and Walker, 1986; Laisk et al., 1989; Rovers and Giersch, 1995), see Roussel and Igamberdiev (2011) for a recent review.

The prototype of most current enzyme kinetic models of the Calvin–Benson cycle was proposed by Pettersson and Ryde-Pettersson (1988). The model is based on mechanistic non-linear enzyme kinetic rate equations, implemented as ODEs, together with equilibrium mass-action ratios. The model describes the dynamics of the Calvin-Benson cycle under conditions of light and CO_2_ saturation. The parameterization of the model involved approximately 50 kinetic parameters, sourced from the literature across several plant species. The model of Pettersson and Ryde-Pettersson (1988), like many current and past kinetic models, is therefore not necessarily a model of a single plant species, but must be considered as a prototype model that describes several generic aspects of plant leaf C3 metabolism. The model was later adapted to investigate further aspects of metabolic regulation in phototrophic metabolism (Poolman et al., 2000, 2001), and extended by Zhu et al. (2007) to investigate the reallocation of enzymes of photosynthetic carbon metabolism with respect to optimal nitrogen and protein investment. These models also served as a blueprint for the first detailed kinetic models of cyanobacterial core carbon metabolism. Jablonský et al. (2013) proposed a modified version of the model of Zhu et al. (2007), adapted to describe the cyanobacterium *Synechococcus elongatus* PCC 7942, to investigate the functional consequences of isoenzymes. The majority of parameters were retained from the original models. The model was later refined (Jablonský et al., 2014) to explain the metabolic regulation of primary carbon metabolism, also incorporating transcriptional data as a constraint for model dynamics. Most recently, a kinetic model of the central carbon metabolism of the cyanobacterium *Synechocystis* sp. PCC 6803 was developed to investigate the role of isozymes on metabolic network homeostasis with respect to changes in gene expression induced by different CO_2_ conditions (Jablonský et al., 2016). In particular, a comparison of model properties indicated that the higher number of isozymes present in the *Synechocystis* sp. PCC 6803 genome compared to the (smaller) genome of *Synechococcus elongatus* PCC 7942 may correspond to a shift of metabolic regulatory strategies from transcriptional control in latter toward post-transcriptional control in the former (Jablonský et al., 2016).

From computational perspective, the kinetic models considered above share several features relevant to the integration into multiscale models of phototrophic growth. In each case, the dynamics of the concentrations of metabolic intermediates are described by ordinary differential equations (ODEs). Rate equations are derived from enzyme kinetic mechanisms, and implemented using (usually reversible) non-linear Michaelis–Menten type functions. The rate equations consider allosteric regulations, as well as other post-translational mechanisms, as far as such interactions are known. The light reactions are typically highly simplified. In the model of Pettersson and Ryde-Pettersson (1988) and its later adaptations, ATP is provided by a single overall reaction (an ATP synthetase) that can be modulated according to light intensity. The concentrations of NADP^+^ and NADPH are assumed to be constant. Likewise, enzyme amounts are represented by external parameters, the respective values are part of the maximal reaction velocities V_max_. Table 2 lists selected kinetic models of central carbon metabolism and the Calvin–Benson cycle. We conjecture that such models provide a reasonable account of metabolite dynamics on short and medium time scales (minutes to few hours) and to metabolic adaptations to brief periods of darkness. As yet, the construction of kinetic models to adequately represent changes in day/night metabolism remains a considerable challenge.

**Table 2:** **Selected kinetic models of the central carbon metabolism in photosynthetic organisms**.

Farquhar et al. (1980)	**A biochemical model of photosynthetic CO_2_ assimilation in leaves of C3 species** An influential early model to describe photosynthetic CO_2_ assimilation *in vivo*. The model describes limiting processes in the leaf and integrates these into overall systems behavior.
Hahn (1984)	**A mathematical model of leaf carbon metabolism** Among the first kinetic models of plant leaf carbon metabolism. The model is based on ODEs for 19 metabolic intermediates using mass action kinetics.
Hahn (1987)	**A mathematical model of photorespiration and photosynthesis** An extension of the earlier ODE model of Hahn (1984) that incorporates photorespiration (glycolate and glycerate pathways) and diffusion between atmosphere and mesophyll tissue using 31 variables.
Pettersson and Ryde-Pettersson (1988)	**A mathematical model of the Calvin photosynthesis cycle** The prototype of most current kinetic models of plant leaf metabolism. The model is based on ODEs and makes use of derived enzyme kinetic rate equations for 9 reactions. 11 reactions are assumed to be close to equilibrium and are represented by algebraic equations within the model. Parameters are sourced from the biochemical literature.
Laisk et al. (1989)	**A mathematical model of the carbon metabolism in photosynthesis. Difficulties in explaining oscillations by fructose 2,6-bisphosphate regulation** The model is similar in scope to the model of Pettersson and Ryde-Pettersson (1988) and provides a detailed description of core carbon metabolism (the Calvin–Benson cycle, starch and sucrose synthesis) involving 20 ODEs. The light reactions are described by a single enzyme-catalyzed reaction, the ATP synthetase. The NADPH/NADP^+^ ratio is assumed to be constant.
Poolman et al. (2000)	**Modeling photosynthesis and its control** An adaptation of the model of Pettersson and Ryde-Pettersson (1988). The major difference is that reactions close to equilibrium are represented by reversible mass action kinetics rather than algebraic equations. The model was later analyzed with respect to its control properties and shows evidence for two steady states (Poolman et al., 2001).
Zhu et al. (2007)	**Optimizing the distribution of resources between enzymes of carbon metabolism can dramatically increase photosynthetic rate: a numerical simulation using an evolutionary algorithm** An extended kinetic model of plant leaf metabolism based on the model of Pettersson and Ryde-Pettersson (1988) including a detailed representation of photorespiratory metabolism. The model was used to study the optimal partitioning of enzymes to increase photosynthetic rate.
Jablonský et al. (2013)	**Phosphoglycerate mutases function as reverse regulated isoenzymes in *Synechococcus elongatus* PCC 7942** A modified and corrected model for cyanobacterial central carbon metabolism, based on the models of Pettersson and Ryde-Pettersson (1988) and Zhu et al. (2007). The model is the first kinetic model specific for a cyanobacterium and was used to investigate the function of phosphoglycerate mutase isoforms in *Synechococcus elongatus* PCC 7942. The model was later improved and extended (Jablonský et al., 2014) to investigate the diverse roles of isoenzymes in the same strain.
Jablonský et al. (2016)	**Different strategies of metabolic regulation in cyanobacteria: from transcriptional to biochemical control** A model of the central carbon metabolism of *Synechocystis* sp. PCC 6803 based on the earlier model of Jablonský et al. (2014). The model describes the dynamics of 36 metabolite concentrations interconnected by 54 reactions and makes use of 182 kinetic parameters.

*The models of Laisk et al. (2006) and Zhu et al. (2013) integrate the photosynthetic light reactions and the core carbon metabolism and are already listed in Table 1. Minimal models that investigate oscillatory mechanisms, and models that primarily focus on plant-specific properties, such as stomatal conductance, are not considered*.

The key factors to integrate models of core carbon metabolism into overall models of phototrophic growth requires an explicit representation of energy (ATP) and redox state (NADPH/NADP^+^) as dynamic variables that allow coupling to the ETC. In this respect, first steps have been made for plant metabolism. The models of Laisk et al. (2006) and Zhu et al. (2013) both integrate the photosynthetic light reactions with a detailed representation of the core C3 carbon metabolism, including photorespiration. Such models provide a framework for guiding engineering efforts and allow for a description of photosynthesis and carbon fixation in response to, for example, changes in photon flux density.

Beyond the integration of light capture and carbon metabolism, significant challenges remain. Allosteric post-translation regulation only covers small to medium time scales. No current kinetic model provides a description of diurnal changes in metabolism and the corresponding switch from carbon assimilation to the mobilization of storage compounds. In addition to redox regulation, such switches are likely to require the inclusion of additional hierarchies of cellular regulation, in particular transcription and possibly regulation by the circadian clock. Switches in metabolic modes are of particular relevance for cyanobacterial metabolism and growth, as they lack the compartmentation of eukaryotic algae and plants. Likewise, current models focus on carbon metabolism and its regulation, limitation of other macronutrients like phosphorus or nitrogen is typically not considered. Nonetheless, in particular with respect to nitrogen, kinetic models can be expected to provide insight into the role of certain metabolites, such as 2-oxoglutarate (2-OG), as signaling compounds (Fokina et al., 2010).

A major obstacle for detailed kinetic models also remains the scarcity of enzyme kinetic data. The construction of kinetic models requires detailed knowledge of enzyme kinetic parameters—with typically 4–5 parameters per reaction, including the Michaelis–Menten parameters *K*_M_ for substrates and products, as well as thermodynamic equilibrium values K_eq_ and the specific catalytic activities of enzymes. Even metabolic pathways of moderate size, such as the Calvin–Benson cycle and adjacent reaction, typically consist of 20–30 enzymatic reactions. Therefore, the construction of larger kinetic models, while feasible from a computational point of view, is primarily limited by data availability and data reliability (Srinivasan et al., 2015). To some extent, the scarcity of information about kinetic parameters can be alleviated by explicitly accounting for uncertainty in kinetic models of metabolism—suitable approaches have been proposed recently (Wang et al., 2004; Steuer et al., 2006; Tran et al., 2008; Steuer and Junker, 2009; Murabito et al., 2014) but are not yet widely applied in models of phototrophic growth.

## 5 Models of Carbon-Concentrating Mechanisms

**Figure 5:**
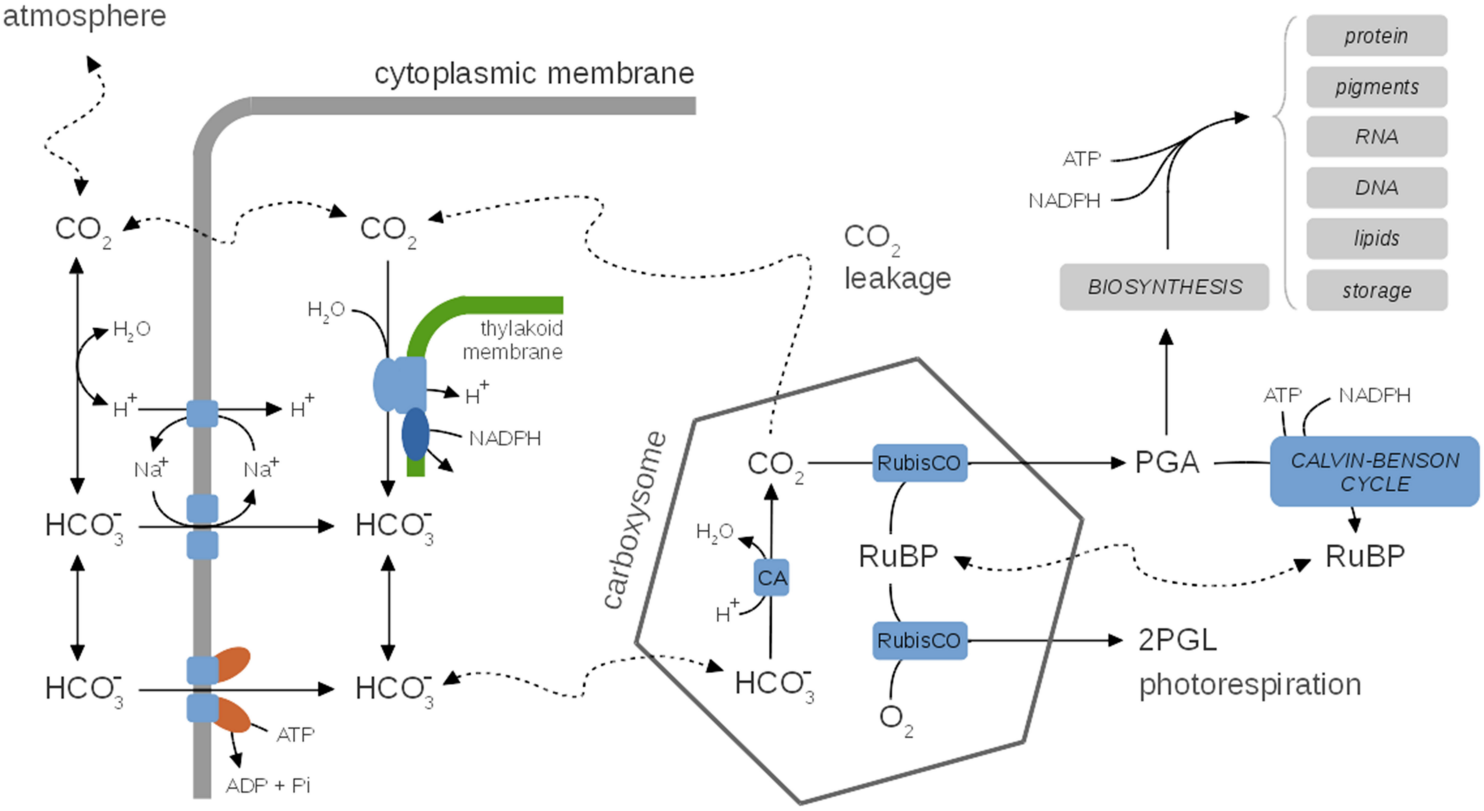
**A generic representation of cyanobacterial CO_2_-concentrating mechanisms (CCM), species-specific differences are neglected**. Inside, the carboxysome bicarbonate $\left({\text{HCO}}_{3}^{-}\right)$ is rapidly converted to CO_2_ via the carbonic anhydrase (CA). CCMs thereby facilitate a CO_2_ gradient to increase the local concentration of CO_2_ in the vicinity of RuBisCO and depress photorespiration. Several uptake mechanisms for ${\text{HCO}}_{3}^{-}$ exist, including ABC-type high affinity transporters and Na^+^/${\text{HCO}}_{3}^{-}$ symporters. The activity of CCMs, including expression of several components, is modulated by environmental parameters, in particular CO_2_ availability. CO_2_ leaking from the carboxysomes may diffuse into the medium or is partly converted back to ${\text{HCO}}_{3}^{-}$ (carbon cycling) at the thylakoid membrane using insufficiently understood mechanisms. CCMs utilize cellular energy and might be involved in dissipating excess light energy and play a role in the maintenance of internal pH. Hence, CCMs are integrally tied to cellular metabolism and growth. See Table 3 for selected computational models of CCMs.

A characteristic feature of cyanobacterial growth is the use of CO_2_-concentrating mechanisms (CCMs) to facilitate the uptake and acquisition of inorganic carbon. CCMs allow cyanobacteria to raise the local concentration of CO_2_ in the vicinity of the carboxylating enzyme RuBisCO, and thereby overcome the comparatively low affinity of RuBisCO for CO_2_ and depress the competitive oxygenation reaction (photorespiration). Cyanobacterial CCMs typically make use of dedicated microcompartments, the carboxysomes, that separate the assimilation of CO_2_ by RuBisCO from the rest of the cell. The CCMs of cyanobacteria relies on a number of components. The respective mechanisms are reasonably well understood (Kaplan and Reinhold, 1999; Price et al., 2008; Burnap et al., 2015), see Figure 5 for a schematic depiction, and the requirement of a quantitative mathematical analysis has recently been highlighted (Mangan and Brenner, 2014). The efficiency of the cyanobacterial CCMs can be characterized by the ratio between the apparent whole-cell affinity for extracellular CO_2_ and the respective affinity for CO_2_ of the carboxylating enzyme RuBisCO—with ratios up to 1,000 reported in the literature (Burnap et al., 2015). While many components of the CCMs are constitutively expressed, the expression of specific uptake systems is differentially regulated depending on environmental parameters, in particular light intensity and the availability of inorganic carbon (Ci) (Kaplan and Reinhold, 1999; Burnap et al., 2015).

In addition to their important function to enhance the local CO_2_ concentration and depressing photorespiration, CCMs may also be involved in dissipating excess light energy (Xu et al., 2008) and might play a role in pH homeostasis. Despite this tight integration with carbon metabolism, none of the current kinetic models of carbon metabolism explicitly accounts for CCMs. Nonetheless, a number of quantitative models of CCMs are available that can be readily incorporated into models of cyanobacterial growth. See Table 3 for selected computational models of CCMs. Early models focus either on simple equations for CO_2_ and ${\text{HCO}}_{3}^{-}$ (bicarbonate) flux into and out of the cell (Badger et al., 1985), as well as on arguments based on reaction–diffusion equations (Reinhold et al., 1987, 1991). These models were refined further to include explicit representations of the carboxysomes. Specifically, the model of Fridlyand et al. (1996) considers the various CO_2_ or ${\text{HCO}}_{3}^{-}$ fluxes between medium, periplasmic space, cytoplasm and carboxysomes using derived values for geometric parameters, and permeability and diffusion coefficients. The model also considers the energetic consequences of scavenging CO_2_ that leaks back into the cytoplasm. Models can be adopted to specific cyanobacterial strains, such as the model of Hopkinson et al. (2014) for *Prochlorococcus* spp. MED4. Two recent quantitative models of CCM functioning are available (Clark et al., 2014; Mangan and Brenner, 2014). Both models are based on reaction–diffusion equations that are solved for highly simplified spatial topologies (spherical cells), but otherwise make use of partially divergent assumptions. The focus of Clark et al., 2014 are interspecies differences and a hypothetical carboxysome-free mutant that is of interest in industrial settings with elevated CO_2_ supply. The model assumes that the carboxysome walls are impermeable to CO_2_. The model of Mangan and Brenner (2014) assumed that carboxysome permeability is identical for ${\text{HCO}}_{3}^{-}$ and CO_2_ and the model explores the range of best parameter values that give rise to a functional and effective CCM. While carboxysome permeability has not yet been measured directly, Mangan and Brenner (2014) concluded that optimal parameter values indeed exist, and transport rates and concentrations derived for these optimal values are in good agreement with known experimental data. Very recently, the model was extended to incorporate the effect of intracellular pH as a key physiological parameter that governs the composition of the Ci pool (Mangan et al., 2016). The “pH-aware” model highlights the utility of quantitative models to evaluate the energetic costs of Ci accumulation for CCMs.

**Table 3:** **Selected models of cyanobacterial CO_2_-concentrating mechanisms (CCMs)**.

Badger et al. (1985)	**A model for ${\text{HCO}}_{3}^{-}$ accumulation and photosynthesis in the cyanobacterium *Synechococcus* sp: theoretical predictions and experimental observations** A simple early model of CCMs in cyanobacteria based on a single spherical compartment into which inorganic carbon is actively accumulated.
Fridlyand et al. (1996)	**Quantitative evaluation of the role of a putative CO_2_-scavenging entity in the cyanobacterial CO_2_-concentrating mechanism** Based on a series of earlier models (Reinhold et al., 1991), the model provides a quantitative account of cyanobacterial CCM with a focus on the implications of scavenging CO_2_ that leaks outwards from the carboxysome. The model is based on eight differential equations that describe diffusion and transport of inorganic carbon (CO_2_ and${\text{HCO}}_{3}^{-}$) between the various compartments.
Hopkinson et al. (2014)	**The minimal CO2-concentrating mechanism of *Prochlorococcus* spp. MED4 is effective and efficient** A simple model based on the model of Reinhold et al. (1987) and adapted from *Prochlorococcus* spp. MED4. Transfer of inorganic carbon is described by four differential equations.
Clark et al. (2014)	**Insights into the industrial growth of cyanobacteria from a model of the carbon-concentrating mechanism** A kinetic model of the CCM parameterized for two cyanobacterial species and a hypothetical no-CCM mutant. The work assumes the carboxysome shell to be impermeable to CO_2_ and concludes that carboxysome geometry is unimportant and interspecies differences in CCMs are largely due to active ${\text{HCO}}_{3}^{-}$ transporters.
Mangan and Brenner (2014)	**Systems analysis of the CO_2_-concentrating mechanism in cyanobacteria** A quantitative evaluation of cyanobacterial CCMs based on a reaction–diffusion model to investigate parameter ranges that give rise to a functional and effective CCM.
Mangan et al. (2016)	**pH determines the energetic efficiency of the cyanobacterial CO_2_-concentrating mechanism** An update of the model of (Mangan and Brenner, 2014) that introduces intracellular pH as a key physiological parameter that determines the energetic costs associated with CCMs and carbon fixation.

*Modeling the CCM typically involves a spatial component. Analysis of the respective partial-differential equations (PDEs) shows, however, that intracompartmental concentration gradients can be neglected. Models of the CCM are therefore typically based on ODEs, rather than PDEs*.

While current models of the CCM do consider the optimality and functioning of CCMs under different intracellular and environmental conditions, they typically do not incorporate explicit models of cellular growth or other cellular mechanisms. However, the high energy demand of Ci transport, either by direct hydrolysis of one ATP per bicarbonate, or indirectly via the costs of ion transport, the costs of synthesizing carboxysome shell proteins, as well as the significant impact of CCMs on the efficiency of carbon assimilation suggest that further integration of models of CCMs into a broader context of cellular functioning is worthwhile to understand the trade-offs and interactions between energy investment, CCM utilization, carbon assimilation, and growth.

## 6 Large-Scale Models of Cyanobacterial Metabolism

Beyond kinetic models of central carbon metabolism, metabolic networks are increasingly described using large-scale stoichiometric reconstructions (Steuer et al., 2012). Metabolic reconstructions aim to provide a comprehensive account of all possible interconversions of small molecules within a given cell or compartment, including enzymatic reactions as well as non-catalyzed (spontaneous) interconversions, transport reactions, and diffusion. Metabolic reconstructions are derived from annotated genomes with subsequent steps of manual curation and gap filling, see Knoop et al. (2010), Steuer et al. (2012), and Knoop et al. (2013) for recent examples. The description typically involves only knowledge about the stoichiometry of interconversions; knowledge about kinetic parameters (such as Michaelis–Menten parameters) or allosteric regulation is not required.

Nonetheless, large-scale stoichiometric models of bacterial metabolism are highly predictive (McCloskey et al., 2013). The predictive power derives from the fact that the fluxes through enzymatic reactions are not independent. Constraint-based computational methods rely on the fact that under steady-state conditions most intracellular metabolites do not accumulate. The rate of synthesis of any non-accumulating metabolite must therefore approximately equal the rate of consumption of this metabolite. Similar arguments also hold for diurnal metabolism: if, after a full diurnal cycle, the concentration of a given intracellular metabolite is approximately equal to its initial value, then the total flux of synthesis reactions and the total flux of consuming reactions must be approximately equal. The condition of flux balance puts significant constraints on the feasible flux space. Predictions about specific flux solutions are then typically based on assumptions about metabolic optimality. That is, among all feasible flux solutions, constraint-based methods seek to identify a solution that maximizes a given objective function, such as the maximal yield of biomass for a given light input—motivated by the fact that a similar selection might take place during evolution. Predictions from large-scale stoichiometric models are therefore not mechanistic, that is, they are not derived from knowledge about biophysical or biochemical interactions. Rather, predictions are derived from how metabolism *ought* to behave given the assumption that metabolic functioning fulfills certain evolutionary optimality principles.

Computationally, the analysis of large-scale stoichiometric reconstructions is based on methods of linear programing (LP) and is computationally feasible also for networks consisting of several thousands of reactions. The strength of stoichiometric models and constraint-based analysis are questions such as the following: What is the maximal growth yield for a given light or carbon input? Which set of enzymes is essential for the synthesis of certain biomass components? How many distinct biochemical paths exist for the synthesis of certain biomass components and how do these pathways differ with respect to cellular energy expenditure and cofactor utilization? Due to the specific computational methodology, however, a direct integration of large-scale stoichiometric models into kinetic models of metabolism remains challenging. Various extensions toward incorporating dynamics have been proposed (Mahadevan et al., 2002; Kim et al., 2008; Feng et al., 2012; Antoniewicz, 2013), and extensive efforts are undertaken to bridge the gap between kinetic and stoichiometric models (Steuer, 2007; Steuer and Junker, 2009; Chakrabarti et al., 2013; Srinivasan et al., 2015).

Detailed stoichiometric reconstructions are available for several cyanobacterial strains (Knoop et al., 2010, 2013; Montagud et al., 2010; Hamilton and Reed, 2012; Nogales et al., 2012; Saha et al., 2012; Vu et al., 2012; Mueller et al., 2013; Maarleveld et al., 2014; Yoshikawa et al., 2015)—typically consisting of several hundred enzymatic interconversions and accounting for all known pathways related to central metabolism and the synthesis of key biomass components. See Table 4 for selected examples of metabolic reconstructions. Such large-scale reconstructions are valuable tools to derive consistent equations for the (maximal) growth yield with respect to light input, to derive core models of reaction pathways, and to make predictions about maximal product yield in biotechnological applications (Zavřel et al., 2016). In particular, large-scale reconstructions also enable a semiautomated extraction of meaningful core models to facilitate the construction of smaller kinetic models (Erdrich et al., 2015). Analysis of the respective networks, however, is often confined to either a constant light environment, or to heterotrophic growth on extracellular carbon sources. Only recently stoichiometric analysis of phototrophic metabolism explicitly described different phases of light availability. For example, Knoop et al. (2013) simulated biomass synthesis fluxes over a full diurnal cycle, Muthuraj et al. (2013) used dynamic FBA (dFBA) to capture light-dark metabolism over discretized time intervals, Knies et al. (2015) considered storage metabolites that accumulate and are consumed over a diurnal cycle using a reconstruction of the unicellular alga *Emiliania huxleyi*, Cheung et al. (2014) described a flux balance model that captures interactions between light and dark metabolism in C3 and CAM leaves, and Baroukh et al. (2014) proposed a novel dynamic modeling framework to describe carbon metabolism of unicellular microalgae.

**Table 4:** **Selected large-scale metabolic reconstructions of cyanobacteria**.

Shastri and Morgan (2005)	**Flux balance analysis of photoautotrophic metabolism** One of the first metabolic reconstruction and stoichiometric evaluations of cyanobacterial central carbon metabolism. The model is medium scale with a focus on central metabolism and does not include gene–reaction associations.
Knoop et al. (2010)	**The metabolic network of *Synechocystis* sp. PCC 6803: systemic properties of autotrophic growth** A large-scale (but not genome-scale) reconstruction of *Synechocystis* sp. PCC 6803 including gene–reaction associations and photorespiration. The model includes the GABA shunt to close the (incomplete) TCA cycle.
Montagud et al. (2010)	**Reconstruction and analysis of genome-scale metabolic model of a photosynthetic bacterium** A reconstruction of *Synechocystis* sp. PCC 6803. The model also includes a putative glyoxylate shunt that subsequently could not be identified *in vivo*.
Saha et al. (2012)	**Reconstruction and comparison of the metabolic potential of cyanobacteria *Cyanothece* sp. ATCC 51142 and Synechocystis sp. PCC 6803** A detailed reconstruction of the cyanobacterium *Cyanothece* sp. ATCC 51142. The model considers separate (light/dark) biomass equations to reflect the differences between light and dark phases.
Vu et al. (2012)	**Genome-scale modeling of light-driven reductant partitioning and carbon fluxes in diazotrophic unicellular cyanobacterium *Cyanothece* sp. ATCC 51142** A reconstruction of *Cyanothece* sp. ATCC 51142 with a focus on relative fluxes through the ETC.
Nogales et al. (2012)	**Detailing the optimality of photosynthesis in cyanobacteria through systems biology analysis** An expanded reconstruction of *Synechocystis* sp. PCC 6803 including gene–reaction associations. The model does not yet incorporate the TCA bypass identified by Zhang and Bryant (2011).
Knoop et al. (2013)	**Flux balance analysis of cyanobacterial metabolism: the metabolic network of *Synechocystis* sp. PCC 6803** An expanded reconstruction of *Synechocystis* sp. PCC 6803, including the TCA bypass (Zhang and Bryant, 2011) as well as the original GABA shunt. The model is backed by experimental analysis to identify or disprove the existence of a functional glyoxylate shunt and considers diurnal changes in reaction fluxes.
Mueller et al. (2013)	**Rapid construction of metabolic models for a family of cyanobacteria using a multiple source annotation workflow** Genome-scale reconstructions of several *Cyanothece* strains that demonstrate the advantages of using a parallel workflow.

*Reconstructions of algae and land plants are not considered*.

Beyond conventional FBA and related constraint-based methods, there has been increasing interest to evaluate cellular metabolism in terms of a cellular “protein economy” (Molenaar et al., 2009) and to study trade-offs in cellular resource allocation (Goel et al., 2012; Müller et al., 2015)—a theme where, as noted by Schaechter (2015), the bacterial growth physiology of old is connected to the systems biology of today (Stouthamer, 1973; Neidhardt et al., 1990). As one of the first applications involving cyanobacteria, Burnap (2015) formulated a model of autotrophic growth in terms of allocating protein resources among core functional groups, such as the ETC, light-harvesting antennae, and ribosomes. Along similar lines, Rügen et al. (2015) formulated a self-consistent autocatalytic model of phototrophic growth. The model is based on the observation that the macromolecules that constrain cellular growth, including the components of the ETC, metabolic enzymes, and ribosomes, are itself products of metabolism. Phototrophic growth can therefore be formulated as a time-dependent linear optimization problem, such that optimal growth entails a time-dependent allocation of resources during a full diurnal cycle. The approach of Rügen et al. (2015), denoted as conditional FBA, results in dynamic time courses for all involved reaction fluxes, as well as changes in biomass composition over a diurnal cycle. Similar to conventional FBA, models of this kind are not based on mechanistic insight, but rather seek to evaluate the optimality of resource allocation during phototrophic growth. It is expected that methods and applications that go beyond conventional FBA and involve spatial and temporal metabolic modeling based on genome-scale reconstructions of microbial metabolism will play an increasingly important role (Henson, 2015).

## 7 Models of the Cyanobacterial Clock

**Figure 6:**
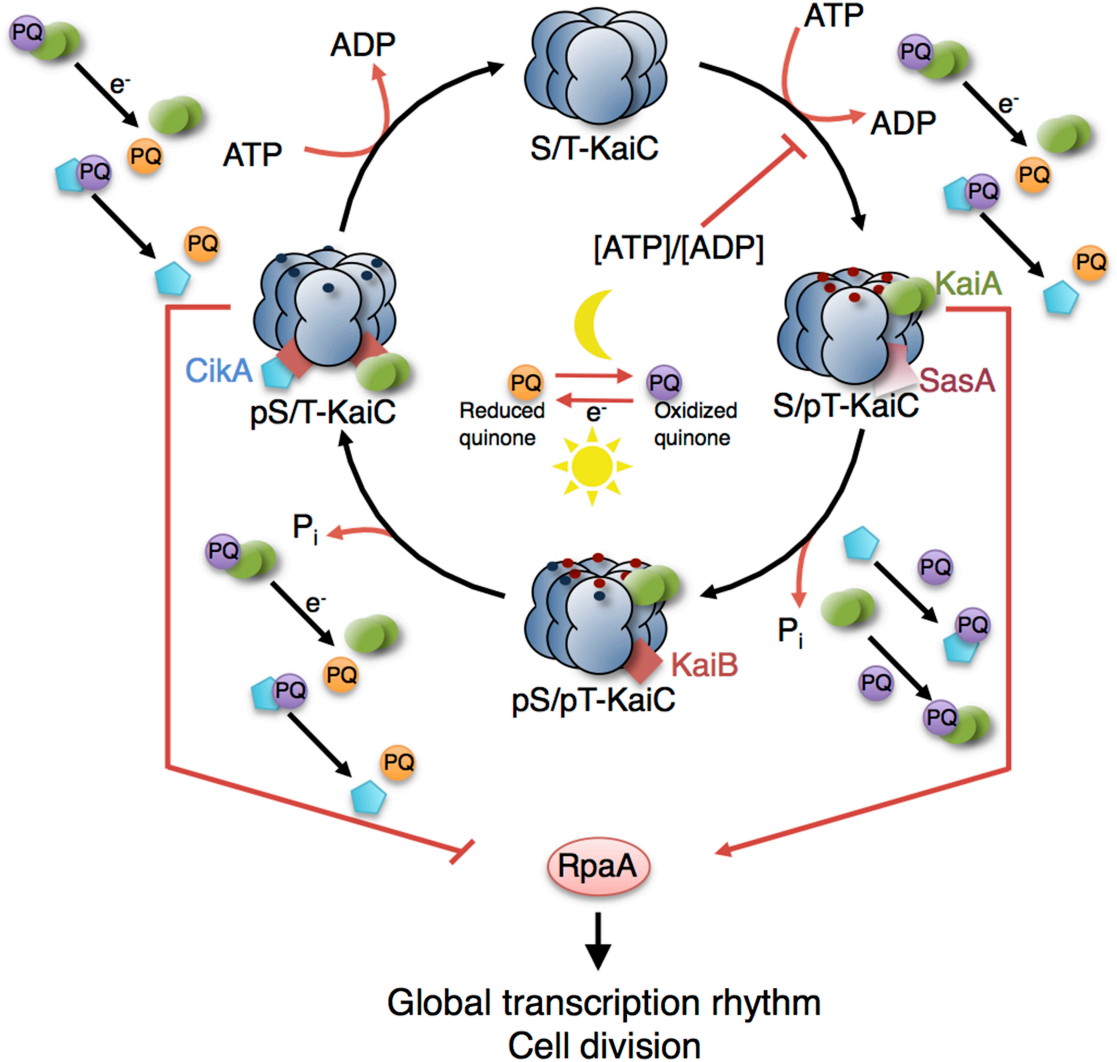
**Model of the circadian clock and putative interaction sites with other metabolic processes**. The KaiC phosphorylation cycle and the KaiABC complex formation are dependent on ATP. Cellular metabolic signals encoded in the ATP/ADP ratio have a direct effect on the core clock by modulating the KaiC’s kinase activity (Rust et al., 2011; Pattanayak et al., 2015). Increasing ADP levels at night reset the phase of oscillation by inhibiting further KaiC phosphorylation. Oxidation and reduction of plastoquinone (PQ) are controlled by photosynthetic electron transport during the day and by electrons derived from the respiratory ETC in the night. At the transition from day into night, quinones become transiently oxidized capturing KaiA and CikA (Kim et al., 2012). The aggregation of KaiA stops KaiC phosphorylation. The core circadian clock generates rhythms in gene expression and cell division via the global transcriptional factor RpaA. During the day, the physical interaction of SasA with KaiC promotes phosphotransfer to RpaA so that RpaA becomes active. During the night, the physical interaction of CikA with the KaiBC complex inhibits phosphorylation of RpaA so that RpaA becomes inactive (Gutu and OShea, 2013). Red and blue dots are phosphates at KaiC phosphorylation sites Thr432 and Ser431. Red arrows represent interactions with ATP, ADP, and oxidized quinones related to metabolic processes of phototrophic growth. The role of other input components (e.g., LdpA) and output components (e.g., LabA, RpaB) as well as the location of the quinones in the thylakoid membranes are not shown. See Table 5 (*in vitro*) and Table 6 (*in vivo*) for selected models of the cyanobacterial circadian clock.

In addition to the biochemistry of metabolism, phototrophic growth is highly dependent on regulatory networks to coordinate growth and to relay environmental information. To this end, of particular relevance is the cyanobacterial circadian clock—discovered in the late 1980s and unique among prokaryotes (Pattanayak and Rust, 2014). The cyanobacterial clock consists of an interrelated network of multifunctional components functioning in timekeeping, input and/or output mechanisms. *Synechococcus elongatus* PCC 7942 is the cyanobacterium whose clock is currently best studied. The core oscillator comprises only three proteins: KaiA, KaiB, and KaiC (Ishiura et al., 1998). KaiC exhibits an intrinsic kinase, dephosphorylation, and ATPase activity (Nishiwaki et al., 2004; Terauchi et al., 2007; Egli et al., 2012; Nishiwaki and Kondo, 2012). In complex with KaiA and KaiB, KaiC undergoes circadian Thr/Ser phosphorylation (Nakajima et al., 2005; Nishiwaki et al., 2007; Rust et al., 2007) (Figure 6). KaiA promotes and KaiB represses phosphorylation of KaiC (Iwasaki et al., 2002; Kitayama et al., 2003; Xu et al., 2003). The ATPase activity of KaiC is extremely weak (only 15 ATP molecules are consumed per day) and slow, determining the about 24-h period of the clock (Terauchi et al., 2007; Abe et al., 2015; Chang et al., 2015). The circadian rhythm of KaiC phosphorylation runs without transcription–translation and can even operate in a test tube (Nakajima et al., 2005; Tomita et al., 2005). *In vivo*, the KaiABC protein system works as a post-translational oscillator (PTO).

The KaiC phosphorylation rhythm has widely been studied in systems biology, and hence, a variety of mathematical models have been put forward. The first models were rather minimal to explain how sustained oscillations in phosphorylation of KaiC occur, including no intermediate steps of phosphorylation, introducing feedbacks on KaiC phosphorylation or assuming hypothetical states of KaiA, KaiB, and KaiC (Emberly and Wingreen, 2006; Kurosawa et al., 2006; Mehra et al., 2006; Axmann et al., 2007; Mori et al., 2007). Emberly and Wingreen (2006) were the first who showed theoretically that monomer shuffling between KaiC hexamers at specific clock times could explain the robustness and resilience of the circadian clock—a hypothesis stated prior to experimental evidence. Different variations of the concept, monomer shuffling, have afterward been modeled by other groups (Kageyama et al., 2006; Mehra et al., 2006; Mori et al., 2007; Yoda et al., 2007; Eguchi et al., 2008; Nagai et al., 2010). Another hypothesis of how synchrony within the Kai oscillator could be achieved stresses KaiA sequestration into KaiABC complexes (Kurosawa et al., 2006; Clodong et al., 2007; Rust et al., 2007; van Zon et al., 2007; Brettschneider et al., 2010). The consensus view has emerged that both mechanisms work in concert (Qin et al., 2010a). See Table 5 (*in vitro*) and Table 6 (*in vivo*) for selected models of the cyanobacterial circadian clock.

**Table 5:** **Selected *in vitro* models of the cyanobacterial circadian clock**.

Emberly and Wingreen (2006)	**Hourglass model for a protein-based circadian oscillator** An early minimal model to explain sustained oscillations in phosphorylated KaiC. The authors suggest monomer exchange between KaiC hexamers to contribute to the robustness of the clock, prior to experimental evidence of such mechanisms. The model consists of 5 variables and 8 parameters.
Mehra et al. (2006)	**Circadian rhythmicity by autocatalysis** An early minimal model using a two-state approximation (KaiC exists in two phosphorylation states, high and low). Oscillations emerge due to autocatalytic KaiA–KaiC interaction. The model consists of eight variables and 10 parameters.
Mori et al. (2007)	**Elucidating the ticking of an *in vitro* circadian clockwork** The model describes explicitly the kinetics of complex formation and dissociation of KaiC hexamers with *k* phoshorylation sites per monomer. Monomer exchange in phosphorylation and dephosphorylation phase explains robust oscillations. The model uses a stochastic matrix model for simulation of hexamer kinetics resulting in a combinatorial number of variables (4,374 variables for *k* = 2 parameters).
Yoda et al. (2007)	**Monomer-shuffling and allosteric transition in KaiC circadian oscillation** Similar to Mori et al. (2007) but using deterministic equations to simulate the dynamics (56 variables, 34 parameters).
van Zon et al. (2007)	**An allosteric model of circadian KaiC phosphorylation** The model uses 2 phosphorylation states for Kai C, KaiA exhibits different binding affinity for different phospho-forms of KaiC. The model emphasizes sequestration of KaiA in KaiA–KaiB–KaiC complexes. The model consists of 30 variables and 17 parameters.
Clodong et al. (2007)	**Functioning and robustness of a bacterial circadian clock** The minimal model was designed to examine different possible feedback mechanisms to determine what type of feedback generates the most robust rhythms. The ODE model consists of 14 variables and 19 parameters.
Rust et al. (2007)	**Ordered phosphorylation governs oscillation of a three-protein circadian clock** An early ODE model to describes the dynamics of the 4 phosphorylation states of KaiC during a circadian cycle. KaiA is sequestrated by serine phosphorylated KaiC using phenomenological assumptions. The model consists of 3 variables and 13 parameters.
Axmann et al. (2007)	**A minimal circadian clock model** A minimal ODE clock model using a two-state approximation of KaiC. KaiA-dependent phosphorylation is highly non-linear, sequestration of free KaiA by phosphorylated KaiC–KaiB complex favors dephosphorylation. The model was designed to study robustness of oscillations with respect to concerted changes in Kai protein concentration but fails to be invariant against such changes (6 variables, 9 parameters).
Eguchi et al. (2008)	**Mechanism of robust circadian oscillation of KaiC phosphorylation *in vitro*** A similar model as Yoda et al. (2007) and Mori et al. (2007). Focus on monomer shuffling to synchronize oscillations. The model consists of 14 variables and 4 parameters.
Nagai et al. (2010)	**Synchronization of circadian oscillation of phosphorylation level of KaiC *in vitro*** Similar to Mori et al. (2007) with focus on monomer shuffling. The model incorporates collective shifts from tense to relaxed states (14 variables and 11 parameters).
Brettschneider et al. (2010)	**A sequestration feedback determines dynamics and temperature entrainment of the KaiABC circadian clock** An ODE model to describe the dynamics of the four phosphorylation states of KaiC during a circadian cycle including complex formation and monomer shuffling (12 variables, 26 parameters).
Qin et al. (2010a)	**Intermolecular associations determine the dynamics of the circadian KaiABC oscillator** An elaborate model to describe complex formation dynamics. The model consists of 56 variables and 34 parameters.
*In vitro* models including energy status	
Rust et al. (2011)	**Light-driven changes in energy metabolism directly entrain the cyanobacterial circadian oscillator** A modified version of the model of Rust et al. (2007). The model includes inhibition of kinase activity by ADP. The ATP/ADP ratio is an explicit parameter within the model (3 variables, 16 parameters).
Phong et al. (2013)	**Robust and tunable circadian rhythms from differentially sensitive catalytic domains** A modified version of the model of Rust et al. (2011) with another ATPase activity that is insensitive to changes in ATP/ADP. The ATP/ADP ratio is an explicit parameter within the model (6 variables, 25 parameters).

*The models focus on the functioning of the post-translational oscillator (PTO). ATP, NADPH, and redox state are typically not considered as explicit variables. All models are implemented as ODEs, except otherwise noted*.

**Table 6:** **Selected *in vivo* models of the cyanobacterial circadian clock**.

Kurosawa et al. (2006)	**A model for the circadian rhythm of cyanobacteria that maintains oscillation without gene expression** The model is based on a two-state approximation and sequestration of KaiA sequestration as mean of synchronization. The model consists of 6 variables and 18 parameters.
Miyoshi et al. (2007)	**A mathematical model for the Kai protein-based chemical oscillator and clock gene expression rhythms in cyanobacteria** The models couples the TTFL and PTO, fully phosphorylated KaiC promotes *kaiBC* transcription. The model consists of 13 variables and 32 parameters and assumes hypothetical states (not seen in experiments).
Zwicker et al. (2010)	**Robust circadian clocks from coupled protein modification and transcription–translation cycles** A PTO and coupled TTFL–PTO model. The model consists of 30 variables and 37 parameters.
Qin et al. (2010b)	**Coupling of a core post-translational pacemaker to a slave transcription/translation feedback loop in a circadian system** An ODE model that describes complex formation dynamics. The model neglects site-dependent phosphorylation and incorporates negative regulation of *kaiBC* transcription by KaiABC and KaiBC complexes. The model consists of 57 variables and 39 parameters.
Hertel et al. (2013)	**Revealing a two-loop transcriptional feedback mechanism in the cyanobacterial circadian clock** An extended version of the model of Brettschneider et al. (2010). The model includes positive and negative regulation of *kaiBC* transcription by threonine and doubly phosphorylated KaiC and unphosphorylated KaiC, respectively (15 variables, 33 parameters).
Teng et al.(2013)	**Robust circadian oscillations in growing cyanobacteria require transcriptional feedback** An extended version of the model of Rust et al. (2007). The model incorporates negative regulation of *kaiBC* transcription by serine phosphorylated KaiC (5 variables, 18 parameters).

*Later models combine the transcription–translation feedback loop (TTFL) and the core PTO oscillator. As for *in vitro* models, ATP, NADPH, and redox state are typically not considered as explicit variables. All models are implemented as ODEs, except otherwise noted*.

Recent studies have increased our understanding of how the core oscillator is integrated with input pathways and output pathways, which enable the clock to synchronize (“entrain”) to the 24-h period of the environment and to transmit temporal information to downstream processes resulting in circadian rhythms in cellular physiology. Clock input cues involve the cellular ATP/ADP ratio, which has a direct effect on the core clock by modulating the KaiCs kinase activity (Rust et al., 2011). In particular, an increase in the ADP levels, occurring when cells are placed into darkness, inhibits further KaiC phosphorylation and thus resets the phase of oscillation to synchronize to the metabolic state of the cell. By simulating various ATP/ADP ratios that mimic different night phases, Rust et al. (2011) could recreate phase shifts in the core oscillator as seen *in vitro* and *in vivo*. For their theoretical analysis, the authors modified a previous mathematical model of the circadian clock, which was based on the rates of phosphorylation and dephosphorylation at Thr432 and Ser431 (Rust et al., 2007). In the refined model, the KaiA-dependent kinase rates were now additionally modulated by the ratio of ATP to ATP + ADP. This model was again adapted to account for an additional ATPase activity experimentally found in the CI subunit of KaiC and required for binding of KaiB to Ser-phosphorylated KaiC (Phong et al., 2013). The KaiBC complex formation was shown to depend on an ATPase, but whose activity was insensitive to changes in the cellular ATP/ADP ratio—in contrast to the ATPase in the CII subunit of KaiC (responsible for the Thr/Ser phosphorylation reactions). The results of the combined modeling and experimental study (Phong et al., 2013) suggest that these two differently sensitive catalytic domains are responsible for the capability of the clock to receive input signals while preserving circadian rhythmicity. Depending on the specific question under investigation, both entrainment models could relatively straightforwardly be integrated with other modules of cyanobacterial physiology, with ATP as key to coupling (e.g., light reactions). Another model version already exists, which additionally accounts for the transcription and translation of clock genes as well as the feedback to the core oscillator but needs to be extended to include interactions with other cellular parameters such as the ATP/ADP ratio (Teng et al., 2013). The mathematical clock model proposed by Brettschneider et al. (2010) could equally be envisaged for the integration into larger models of phototrophic growth. The core oscillator is modeled by a larger set of ODEs (12 ODEs; for comparison, 3 ODEs in Rust et al. (2007) but includes hexamerization of KaiC, monomer shuffling, and assembly and disassembly of KaiAC, KaiBC, and KaiABC complexes. Here also, an extended model (15 ODEs) coupled to a transcription–translation circuit has been proposed (Hertel et al., 2013). Inhibition of KaiCs’ kinase activity by ADP can be incorporated into the model.

Sensing the decrease in the ATP/ADP ratio during night is assumed to allow the clock to infer the length of night (Rust et al., 2011). In two biochemical studies, Pattanayak et al. uncovered a further connection between the clock and cellular metabolism: metabolic rhythms produced by the clock (such as rhythms in glycogen abundance, which go along with changing levels of ATP/ADP) feed back to the core oscillator. These rhythms are very likely the main driving force of the clock, allowing the cells to anticipate the onset of darkness in advance (Pattanayak et al., 2014, 2015). In addition, the clock seems to be able to anticipate nightfall through the plastoquinone pool, which is part of the ETC. In particular, the plastoquinone pool embedded within the thylakoid membrane becomes transiently oxidized at the transition from day into night and binding of PQ to KaiA causing aggregation and decay that, in turn, reduces the positive effect of KaiA on KaiC phosphorylation (Wood et al., 2010; Kim et al., 2012). Other redox-sensitive input components such as CikA (circadian input kinase) and LdpA (light-dependent period A) have been identified, which reset or modulate the phase of KaiC phosphorylation cycle [reviewed by Mackey et al. (2011)]. Figure 6 provides a schematic of possible sites of interactions. Mathematical models describing the interconnections at the molecular level have yet to be developed.

The cyanobacterial circadian clock results in genome-wide gene expression rhythms and regulates cell cycle progression relaying information via a two-component system that comprised SasA (*Synechococcus* adaptive sensor A) and RpaA (regulator of phycobilisome association A) (Liu et al., 1995; Mori et al., 1996; Takai et al., 2006; Dong et al., 2010). Rhythms of chromosome compaction and DNA topology (highly correlated with gene expression rhythms) do not hinge on SasA, pointing to the existence of other output pathways (Smith and Williams, 2006; Woelfle et al., 2007; Vijayan et al., 2009). In the positive transcriptional pathway, SasA interacts physically with KaiC and acts as a kinase toward RpaA (Takai et al., 2006; Gutu and OShea, 2013). In the course of a circadian cycle, KaiB displaces SasA from KaiC and KaiA becomes sequestered, switching KaiC into autodephosphorylation mode (Figure 6). The negative transcriptional output involves LabA (low amplitude and bright), CikA, and the transcriptional factor RpaB (regulator of phycobilisome associated B)—all three repressing the activity of RpaA (Taniguchi et al., 2007; Gutu and OShea, 2013; Espinosa et al., 2015). CikA, with its dual role in input and output pathways, plays a special role. As an output component, CikA competes with KaiA for binding to KaiB (Gutu and OShea, 2013; Chang et al., 2015). The binding of CikA to the KaiBC complex activates the phosphatase activity of CikA toward RpaA (Gutu and OShea, 2013) (Figure 6). RpaA, as both a circadian transcriptional activator and a repressor, drives global gene expression rhythms. The transcriptional output includes the regulation of clock genes, forming a transcription–translation feedback loop (TTFL) to the core oscillator (Ishiura et al., 1998). Since we are just beginning to understand the mechanistic details of the TTFL, the existing mathematical models are still highly simplified, using phenomenological assumptions as to how RpaA (Zwicker et al., 2010) or specific phospho-forms of KaiC control transcription of the *kaiBC* gene cluster (Miyoshi et al., 2007; Qin et al., 2010b; Hertel et al., 2013; Teng et al., 2013). These models reproduce the most important experimental results, although Miyoshi et al. (2007) assumed hypothetical states for KaiA, KaiB, and KaiC inconsistent with experiments. Due the relatively small numbers of variables and parameters, the ODE models of Teng et al. (2013) and Hertel et al. (2013) might be most suitable for use in larger models of phototrophic growth but require additional modifications that account for the most recent experimental findings.

## 8 Regulation of Gene Expression in Cyanobacteria

In *Synechococcus elongatus* PCC 7942, the KaiC phosphorylation cycle targets the general transcription apparatus and thereby regulates 30–100% of the transcriptome in circadian fashion, depending on the experimental setup (Liu et al., 1995; Nakahira et al., 2004; Ito et al., 2009; Vijayan et al., 2009; Lehmann et al., 2013). The transcriptional output is regulated by multiple factors such as circadian changes in chromosomal compaction/decompaction (Smith and Williams, 2006) involving oscillations in DNA supercoiling (Woelfle et al., 2007) as well as biochemical cascade pathways that converge to globally acting transcription factors, RpaA and RpaB and so far unknown factors (Taniguchi et al., 2007; Gutu and OShea, 2013; Paddock et al., 2013; Espinosa et al., 2015). Furthermore, it is now clear that small non-protein-coding RNAs (<200 nucleotides) play as both positive and negative regulators crucial roles in gene expression of cyanobacteria (Georg and Hess, 2011). By base-pairing with the target mRNA, small RNA molecules interfere with the ribosome binding site or other sequence stretches, and consequently alter mRNA translation and stability. This mode of regulation might explain why the proportion of cyclic proteins in diverse cyanobacteria is rather uncorrelated to that found in microarray studies (Stöckel et al., 2011; Waldbauer et al., 2012; Guerreiro et al., 2014), because not only transcriptional but also post-transcriptional (small RNA-mediated) mechanisms might be active and modulate or fine-tune the dynamics of regulatory networks. *Synechocystis* sp. PCC 6803 possesses a large number of small non-coding RNAs, and antisense RNAs influence at least 26% of all gene transcripts in this cyanobacterium. There are several hints that the non-coding RNAs fulfill important functions in light–dark acclimation (Georg et al., 2009; Mitschke et al., 2011). A specific functional role could be clarified for some of the antisense RNAs, e.g., IsrR (Dühring et al., 2006; Legewie et al., 2008), as-flv4 (Eisenhut et al., 2012), or PsbAR2 and PsbAR3 mRNA (Sakurai et al., 2012). Yet, many identified RNA regulators still await elucidation of their functional relevance.

## 9 Conclusion: Putting the Parts Together

Almost all cellular functions have evolved in the context of constraints and trade-offs that can only be understood if the respective cellular and environmental context is taken into account. To this end, the construction of computational models of cellular processes not only allows us to study the inner workings of selected processes but also allows us to investigate the emergent properties that arise from interactions between these processes. The trade-offs and interrelations within phototrophic growth are manifold: the energy required for cellular growth is derived from the photosynthetic light reactions, which themselves are a major source of reactive oxygen species (ROS) and therefore require careful balance between different electron transport pathways and alternative electron “valves”. CCMs use energy and have implications for the efficiency of carbon assimilation. The cellular ATP/ADP ratio and the oxidized PQ pool relays information to the circadian clock, which affects transcriptional output and hence metabolic activity. Metabolism itself depends on cellular energy and redox potential—and must be appropriately coordinated to synthesize the right metabolites at the right time. The availability of ribosomes and amino acids, which are itself products of metabolism, affects the rates of translation of new proteins—which must be coordinated to account for damage, stability, and turnover times of proteins. In particular, the components of PSII complexes themselves are dependent on environmental conditions due to photodamage caused by ROS (Yao et al., 2012).

Many if not most of these interactions and trade-offs are still insufficiently understood. An important example is the action and the evolutionary benefit of the circadian clock. While many if not most prokaryotes live in environments with periodic diurnal cycles of light, temperature, and humidity, only cyanobacteria are known to possess a *bona fide* circadian clock. While the competitive advantage of a circadian clock in a periodic environment has been demonstrated experimentally for cyanobacteria (Woelfle et al., 2004), the precise adaptive value and the selective pressure resulting the evolution of a clock remains only partially understood. Reasoning about the possible fitness implications of a circadian clock necessarily involves considering the organisms as a whole, as exemplified in the “escape from light” hypothesis that circadian rhythmicity arose from the need to protect the organism’s DNA from ultraviolet (UV) radiation, at the time unfiltered by the Earth’s early atmosphere (Hut and Beersma, 2011; Lück and Westermark, 2016). A quantitative evaluation of such a hypothesis requires to contrast the energetic cost of the circadian clock with its benefits for survival and growth—a task where advanced computational models will allow for an increasingly quantitative evaluation.

While, as outlined in this contribution, a large number of computational models related to phototrophic growth are already available, also many important cellular processes are still insufficiently described. An important example is the coordination of cellular growth in response to transient darkness, starvation, or stress conditions. Only recently, iconic pathways, such as the stringent response, have been shown to be active also in cyanobacteria and to mediate a coordinated transcriptional and translational reaction to (transient) periods of darkness (Hood et al., 2016). Likewise, knowledge about the molecular and physiological mechanisms involved in the transition of cyanobacterial cells from a resting state to an active vegetative state is still incomplete (Klotz et al., 2016)—albeit crucial to understand what mechanisms causes a cyanobacterial cell to divert resources away from growth and division and toward survival until environmental conditions improve. Also, only little is known concerning the coordination of cellular metabolism with cell cycle events (Asato, 2005, 2006). In particular, cell size control and size homeostasis in bacteria is still not fully understood, with conceptual models dating back to the work of Donachie (1968). Recent work on *E. coli* and *B. subtilis* favored the “ädder” model, in which the size added between birth and division is constant for a given growth condition—as opposed to the “sizer,” in which the cell actively monitors cell size, and “timer” model, in which the cell grows for a specific time before division (Taheri-Araghi et al., 2015). Corresponding studies for cyanobacteria are not yet available. Recent data have indicated that there is coupling between circadian oscillator and the cell cycle, specifically that cell cycle progression in some cyanobacteria slows during specific circadian intervals (Dong et al., 2010; Yang et al., 2010)—posing timely questions for further computational research and necessitating integrative modules of cyanobacterial growth.

Overall, there is increasing interest in whole-cell models to understand cellular trade-offs and functions in the context of a living cells. The construction of integrative computational models to predict phenotype from genotype has gained momentum with a first whole-cell model of the life cycle of the human pathogen *Mycoplasma genitalium*—based on a subdivision of cell functionality into modules (Karr et al., 2012). A similar effort was already undertaken for cyanobacteria to explain fitness advantage conveyed by a circadian clock (Hellweger, 2010)—an approach that can be regarded as a prototype for the path outlined in this contribution.

Distinct from other approaches to whole-cell models, however, we argue that it is unlikely that a single universal model—a model that spans all scales from intracellular to intercellular to properties of ecosystems—will fulfill all requirements needed to describe cellular growth. Rather, we envision a modular approach. Depending on the research question, different temporal and spatial scales must be considered. The task is then to derive an appropriate representation of cellular processes that accounts for the spatial and temporal scales involved. The derived submodels should be consistent with more fine-grained representation and their construction must be informed by knowledge how processes interact and which trade-offs are relevant for a specific research question. We note that, besides the biological challenges, such a strategy also entails major computational challenges. As yet, the annotation of computational models is often poor. That is, the biochemical identity of model variables is not defined in a computer-readable format, and hence, merging of models typically requires extensive manual curation (Krause et al., 2010). While standardized exchange formats for computational models, such as the Systems Biology Markup Language, SBML (Hucka et al., 2003), are available for more than a decade, they are not commonly applied outside the Systems Biology community. For example, as yet, none of the models of the cyanobacterial circadian clock are available from the BioModels database—a major resource for computational models of biological processes (Li et al., 2010). In addition, only few computational tools allow for the integration of different modeling concepts, such as constraint-based and kinetic models.

Notwithstanding these challenges in computational methodology, we expect a growing library of models related to cyanobacterial growth that can inform and guide research how phototrophic organisms, such as cyanobacteria, adapt to complex environments. These models must increasingly adhere to common standards and should be made available on open platforms, such as the BioModels database (Li et al., 2010) and e-cyanobacterium.org (Klement et al., 2013). Given the recent advances in model development and annotation, computational modeling will undoubtedly play a key role in understanding trade-offs and adaptations in cyanobacteria. In the beginning, integrated models of cyanobacterial growth will be still idealistic, crude, and most certainly incomplete. But, again referring to Neidhardt (1999), “it is only through such modeling of whole-system behavior—that is, of growth—that one will learn how near and how far our knowledge takes us toward understanding the living cell.”

## Author Contributions

SW and RS conducted the research and wrote the article. Both authors approved the final manuscript.

## Conflict of Interest Statement

The authors declare that the research was conducted in the absence of any commercial or financial relationships that could be construed as a potential conflict of interest.

## Appendix

The differential equations that define the model shown in Figure 4 are d[PSU*]/d*t* = *v*_1_ − *v*_2_ − *v_d_* and d[PSU*_d_*]/d*t* = *v_d_* − *v_r_*, together with the conservation relationship [PSU] + [PSU*] + [PSU*_d_*] = [PSU_total_]. The reactions rates describe the activation of the photosynthetic unit (PSU), *v*_1_ = *k*_1_⋅*I*⋅[PSU], the return to the open state, *v*_2_ = *k*_2_⋅[PSU*], the transition to a photodamaged state, *v_d_* = *k_d_*⋅*I*⋅[PSU*], and the recovery, *v_r_* = *k_r_*⋅[PSU*_d_*]. The rate of activation, *v*_1_, and photodamage, *v_d_*, both depend on the light intensity, *I* (with *k_d_* ≪ *k*_1_). Parameters used are *k*_1_ = 10.0, *k*_2_ = 10.0, *k_r_* = 1.0, [PSU_total_] = 1.0, and *k_d_* as indicated in Figure 4. The rate of oxygen evolution (*v*_2_) obtained from an (analytical) steady-state solution of the ODEs is shown. Parameters are arbitrary and do not reflect actual values found in cyanobacteria.
